# T-2 toxin induced *Salmonella *Typhimurium intoxication results in decreased *Salmonella *numbers in the cecum contents of pigs, despite marked effects on *Salmonella*-host cell interactions

**DOI:** 10.1186/1297-9716-43-22

**Published:** 2012-03-22

**Authors:** Elin Verbrugghe, Virginie Vandenbroucke, Maarten Dhaenens, Neil Shearer, Joline Goossens, Sarah De Saeger, Mia Eeckhout, Katharina D'Herde, Arthur Thompson, Dieter Deforce, Filip Boyen, Bregje Leyman, Alexander Van Parys, Patrick De Backer, Freddy Haesebrouck, Siska Croubels, Frank Pasmans

**Affiliations:** 1 Department of Pathology, Bacteriology and Avian Diseases, Faculty of Veterinary Medicine, Ghent University, 9820 Merelbeke, Belgium; 2 Department of Pharmacology, Toxicology and Biochemistry, Faculty of Veterinary Medicine, Ghent University, 9820 Merelbeke, Belgium; 3 Department of Pharmaceutics, Faculty of Pharmaceutical Sciences, Ghent University, 9000 Ghent, Belgium; 4 Department of Foodborne Bacterial Pathogens, Institute of Food Research, Norwich Research Park, NR4 7UA Norwich, UK; 5 Department of Bioanalysis, Faculty of Pharmaceutical Sciences, Ghent University, 9000 Ghent, Belgium; 6 Department of Food Science and Technology, Faculty of Applied bioengineering, University College Ghent, 9000 Ghent, Belgium; 7 Department of Basic Medical Sciences, Faculty of Medicine and Health Sciences, Ghent University, 9000 Ghent, Belgium

## Abstract

The mycotoxin T-2 toxin and *Salmonella *Typhimurium infections pose a significant threat to human and animal health. Interactions between both agents may result in a different outcome of the infection. Therefore, the aim of the presented study was to investigate the effects of low and relevant concentrations of T-2 toxin on the course of a *Salmonella *Typhimurium infection in pigs. We showed that the presence of 15 and 83 μg T-2 toxin per kg feed significantly decreased the amount of *Salmonella *Typhimurium bacteria present in the cecum contents, and a tendency to a reduced colonization of the jejunum, ileum, cecum, colon and colon contents was noticed. In vitro, proteomic analysis of porcine enterocytes revealed that a very low concentration of T-2 toxin (5 ng/mL) affects the protein expression of mitochondrial, endoplasmatic reticulum and cytoskeleton associated proteins, proteins involved in protein synthesis and folding, RNA synthesis, mitogen-activated protein kinase signaling and regulatory processes. Similarly low concentrations (1-100 ng/mL) promoted the susceptibility of porcine macrophages and intestinal epithelial cells to *Salmonella *Typhimurium invasion, in a SPI-1 independent manner. Furthermore, T-2 toxin (1-5 ng/mL) promoted the translocation of *Salmonella *Typhimurium over an intestinal porcine epithelial cell monolayer. Although these findings may seem in favour of *Salmonella *Typhimurium, microarray analysis showed that T-2 toxin (5 ng/mL) causes an intoxication of *Salmonella *Typhimurium, represented by a reduced motility and a downregulation of metabolic and *Salmonella *Pathogenicity Island 1 genes. This study demonstrates marked interactions of T-2 toxin with *Salmonella *Typhimurium pathogenesis, resulting in bacterial intoxication.

## Introduction

T-2 toxin is a type A trichothecene, produced by various *Fusarium *spp. such as *Fusarium acuminatum*, *F. equiseti*, *F. poae *and *F. sporotrichioides *[1]. In moderate climate regions of North America, Asia and Europe, these moulds are common contaminants of cereals such as wheat, barley, oats, maize and other crops for human and animal consumption [2]. Since mycotoxins are very stable under normal food processing conditions, T-2 toxin can end up in the food and feed. With T-2 toxin being the most acute toxic trichothecene [3], this mycotoxin may pose a threat to human and animal health around the world. Pigs appear to be one of the most sensitive species to *Fusarium *mycotoxins [4]. Moderate to high levels of T-2 toxin cause a variety of toxic effects including immunosuppression, feed refusal, vomiting, weight loss, reduced growth and skin lesions [5]. Only little information is available on in vivo effects from humans with known exposure to T-2 toxin. Wang et al. reported an outbreak of human toxicosis in China caused by moldy rice contaminated with T-2 toxin at concentrations ranging from 180 to 420 μg T-2 toxin per kg, and the main symptoms were nausea, vomiting, abdominal pain, thoracic stuffiness and diarrhea [6]. Furthermore, it is suggested that alimentary toxic aleukia (ATA), which occurred in the USSR in the period 1941-1947, is related to the presence of T-2 toxin producing *Fusarium *spp. in over-wintered grain. Clinical symptoms include inflammation of gastric and intestinal mucosa, leukopenia, hemorrhagic diathesis, granulopenia, bone marrow aplasia and sepsis [7]. Although a tolerable daily intake (TDI) value for the sum of T-2 toxin and HT-2 toxin of 100 ng/kg has been set by the European Union [8], control of exposure is limited since no maximum guidance limits for T-2 toxin in food and feedstuff are yet established by the European Union. However, contamination of cereals with T-2 toxin is an emerging issue and concentrations up to 1810 μg T-2 toxin per kg wheat have been reported in Germany [9].

Besides mycotoxins, *Salmonella enterica *subspecies *enterica *serovar Typhimurium (*Salmonella *Typhimurium) infections are a major issue in swine production and one of the major causes of foodborne salmonellosis in humans [10]. Pigs infected with *Salmonella *Typhimurium mostly carry this bacterium asymptomatically in their tonsils, gut and gut-associated lymphoid tissue for weeks or even months [11]. These carrier pigs excrete very low numbers of *Salmonella *and are difficult to distinguish from uninfected pigs. However, at slaughter they can be a source of environmental and carcass contamination, leading to higher numbers of foodborne *Salmonella *infections in humans. Although nontyphoidal *Salmonella *infections in humans mostly result in gastroenteritis, it is still a major cause of morbidity and mortality worldwide. It is estimated that nontyphoidal *Salmonella *infections result in 93.8 million illnesses globally each year, of which 80.3 million are foodborne, and 155 000 result in death [12].

T-2 toxin is rapidly absorbed in the small intestine [13] and affects the porcine and human innate immune system at various levels [14,15]. Since the pathogenesis of a *Salmonella *infection is characterized by a systemic and an enteric phase of infection, T-2 toxin might interfere with the pathogenesis of *Salmonella *Typhimurium. However, until now, there are no data available describing an interaction between low concentrations of T-2 toxin and the pathogenesis of a *Salmonella *Typhimurium infection in pigs. Only some scarce results have been reported of an altered susceptibility to intestinal infections after ingestion of sometimes high and even irrelevant concentrations of certain mycotoxins. Feeding pigs with 5 mg T-2 toxin per kg feed, resulted in a substantial increase in aerobic bacterial counts in the intestine [16]. Tai and Pestka showed that the oral exposure of mice to T-2 toxin could result in an impaired murine resistance to *Salmonella *Typhimurium [17]. Furthermore Oswald et al. showed that fumonisin B1 (FB1) increases the intestinal colonization by pathogenic *Escherichia coli *in pigs [18]. However, Tanguy et al. stated that feeding pigs with FB1 did not induce modifications in the number of *Salmonella *bacteria in the ileum, cecum and colon of pigs [19].

With T-2 toxin and *Salmonella *being two phenomenons to which pigs can be exposed during their lives, the aim of the presented study was to investigate the effects of low and in practice relevant concentrations of T-2 toxin on the course of a *Salmonella *Typhimurium infection in pigs and to elucidate if it alters bacterium-host cell interactions.

## Materials and methods

### Chemicals

T-2 toxin (Sigma-Aldrich, Steinheim, Germany) stock solution of 5 mg/mL was prepared in ethanol and stored at -20°C. Serial dilutions of T-2 toxin were prepared in Luria-Bertani broth (LB, Sigma-Aldrich) or in the corresponding cell culture medium, depending on the experiment.

### Bacterial strains and growth conditions

*Salmonella *Typhimurium strain 112910a, isolated from a pig stool sample and characterized previously by Boyen et al., was used as the wild type strain in which the spontaneous nalidixic acid resistant derivative strain (WTnal) was constructed [20]. The construction and characterization of a deletion mutant in the gene encoding the SPI-1 regulator HilA has been described before [21]. Unless otherwise stated, the bacteria were generally grown overnight (16 to 20 h) as a stationary phase culture with aeration at 37°C in 5 mL of LB broth. To obtain highly invasive late logarithmic cultures for invasion assays, 2 μL of a stationary phase culture were inoculated in 5 mL LB broth and grown for 5 h at 37°C without aeration [22].

For oral inoculation of pigs, the WTnal was used to provide a selectable marker for identification of experimentally introduced bacteria when plating tonsillar, lymphoid, intestinal and faecal samples. The bacteria were grown for 16 h at 37°C in 5 mL LB broth on a shaker, washed twice in Hank's buffered salt solution (HBSS, Gibco, Life Technologies, Paisley, Scotland) by centrifugation at 2300 × *g *for 10 min at 4°C and finally diluted in HBSS to the appropriate concentration of 10^7 ^colony forming units (CFU) per mL. The number of viable *Salmonella *bacteria per mL inoculum was determined by plating 10-fold dilutions on Brilliant Green Agar (BGA, international medical products, Brussels, Belgium) supplemented with 20 μg/mL nalidixic acid (BGA^NAL^) for selective growth of the mutant strains.

### Experimental infection with *Salmonella *Typhimurium of pigs fed T-2 toxin- supplemented diets

All animal experiments were carried out in strict accordance with the recommendations in the European Convention for the Protection of Vertebrate Animals used for Experimental and other Scientific Purposes. The experimental protocols and care of the animals were approved by the Ethics Committee of the Faculty of Veterinary Medicine, Ghent University (EC 2010/049 + expansion 2010/101).

#### Experimental design

Three-week-old piglets (commercial closed line based on Landrace) from a serologically *Salmonella *negative breeding herd (according to the Belgian *Salmonella *monitoring program) were used in this in vivo trial. The *Salmonella*-free status of the piglets was tested serologically using a commercially available *Salmonella *antibody test kit (IDEXX, Hoofddorp, The Netherlands), and bacteriologically via multiple faecal sampling. At arrival, the piglets were randomized into three groups of 5 piglets (Table 1) and each group was housed in separate isolation units at 26°C under natural day-night rhythm with ad libitum access to feed and water. The first 6 days after arrival, all piglets received a commercial blank piglet feed (DANIS, Koolskamp, Belgium) that contains all the nutrients for proper growth, as an acclimatisation period. The feed was free from mycotoxin-contamination, as determined by multi-mycotoxin liquid chromatography tandem mass spectrometry (LC-MS/MS) [23] and the composition of the feed is provided in the Additional file 1. The acclimatisation period was followed by a feeding period of 23 days with the experimental feed diets that were prepared by adding T-2 toxin to the blank feed. The first group received ad libitum blank feed (control group), the second group received feed contaminated with 15 μg/kg T-2 toxin (15 ppb group) and the third group received feed contaminated with 83 μg/kg T-2 toxin (83 ppb group). These concentrations were chosen based on previous measurements of T-2 toxin contamination of feed [23]. After a feeding period of 18 days, the pigs were orally inoculated with 2 × 10^7 ^CFU of *Salmonella *Typhimurium WT_nal_. Five days after inoculation, the pigs were euthanized and samples of tonsils, ileocaecal lymph nodes, duodenum, jejunum, ileum, cecum, colon, contents of cecum and colon and rectal faeces were collected for bacteriological analysis to determine the number of *Salmonella *bacteria. To investigate the intestinal cytokine response, ileal fragments were immediately frozen in liquid nitrogen and stored at -70°C until analysis. Furthermore, to determine the average weight gain (%) of the pigs, the animals were individually weighed after the acclimatization period and, in order to exclude a possible effect of the *Salmonella *infection on the weight gain, after a feeding period of 18 days.

**Table 1:** Distribution of the sexes of the pigs that received, during 18 days, blank feed (control group), feed contaminated with 15 μg T-2 toxin per kg feed (15 ppb group) or feed contaminated with 83 μg T-2 toxin per kg feed (83 ppb group), their respective weight at the beginning of the experiment and their average weight gain.

		Distribution of sexes	Weight at beginning of the experiment (kg)	Average weight gain per group per day (kg/day)	Average weight gain during 18 days per group (%)
control group	piglet 1	female	6.5	0.326 ± 0.08	102 ± 17.3
	piglet 2	female	5.5		
	piglet 3	female	5.5		
	piglet 4	male	5.0		
	piglet 5	male	6.0		
15 ppb group	piglet 6	female	4.5	0.322 ± 0.08	104 ± 14.2
	piglet 7	male	7.0		
	piglet 8	female	5.0		
	piglet 9	male	4.5		
	piglet 10	male	7.0		
83 ppb group	piglet 11	female	5.5	0.239 ± 0.04	70.9 ± 11.3*
	piglet 12	male	7.5		
	piglet 13	female	6.0		
	piglet 14	male	6.5		
	piglet 15	male	5.0		

Superscript (*) refers to a significant difference compared to the control group (*p *< 0.05).

#### Bacteriological analysis

All tissues and samples were weighed and 10% (w/v) suspensions were prepared in buffered peptone water (BPW, Oxoid, Basingstoke, United Kingdom). The samples were homogenized with a Colworth stomacher 400 (Seward and House, London, United Kingdom) and the number of *Salmonella *bacteria was determined by plating 10-fold dilutions on BGA^NAL ^plates. These were incubated for 16 h at 37°C. The samples were pre-enriched for 16 h in BPW at 37°C and, if negative at direct plating, enriched for 16 h at 37°C in tetrathionate broth (Merck KGaA, Darmstadt, Germany) and plated again on BGA^NAL^. Samples that were negative after direct plating but positive after enrichment were presumed to contain 83 CFU per gram tissue or contents (detection limit for direct plating). Samples that remained negative after enrichment were presumed to be free of *Salmonella *in 1 gram tissue or contents and were assigned value "1" prior to log transformation. Subsequently the number of CFU for all samples was converted logarithmically prior to calculation of the average differences between the log10 values of the different groups and prior to statistical analysis.

#### Intestinal cytokine response analysis

Total RNA from the intestinal samples was isolated using RNAzol^®^RT (MRC Inc., Cincinnati, USA) according to the manufacturer's instructions. Extracted RNA was resuspended in 20 μL ultra-pure water. The RNA concentration was measured by absorbance at 260 nm using a nanodrop spectrophotometer (Thermo Scientific, Wilmington, USA) and the integrity of the RNA samples was checked using an Experion RNA StdSens Analysis kit (Biorad Laboratories, Hercules, CA, USA). The construction of cDNA and real-time quantitative PCR analysis to quantify IL-1β, IL-6, IL-8, IL-12, IL-18, TNFα, IFNγ and MCP-1, were carried out as described by Vandenbroucke et al. [24].

### Effects of T-2 toxin on host-pathogen interactions between *Salmonella *Typhimurium and porcine host cells

#### Cytotoxicity of T-2 toxin towards *Salmonella *Typhimurium infected porcine macrophages and intestinal epithelial cells

It is possible that T-2 toxin increases the toxicity of *Salmonella *Typhimurium for host cells, resulting in an increased cell death. Therefore, the cytotoxic effect of T-2 toxin on *Salmonella *Typhimurium infected primary porcine alveolar macrophages (PAM) and intestinal porcine epithelial (IPEC-J2) cells was determined using the neutral red (3-amino-7-dimethylamino-2-methyl-phenazine hydrochloride) uptake assay [25]. Both cell cultures were isolated and cultured as previously described [26]. PAM were seeded in a 96-well microplate at a density of approximately 2 × 10^5 ^cells per well and were allowed to attach for 2 h. The IPEC-J2 cells were seeded in a 96-well microplate at a density of approximately 2 × 10^4 ^cells per well and allowed to grow for either 24 h or 21 days, representing actively dividing and differentiated cells respectively. Subsequently, a *Salmonella *gentamicin protection invasion assay was performed as follows. The host cells were inoculated with *Salmonella *at a multiplicity of infection (MOI) of 10:1. To synchronize the infection, the inoculated multiwell plates were centrifuged at 365 × *g *for 10 min and incubated for 30 min at 37°C under 5% CO_2_. Subsequently, the cells were washed 3 times with Hank's buffered salt solution with Ca^2+ ^and Mg^2+ ^(HBSS+, Gibco) and fresh medium supplemented with 100 μg/mL gentamicin (Gibco) was added. Following a 1 h incubation, the medium was replaced by fresh medium containing 20 μg/mL gentamicin whether or not supplemented with different concentrations of T-2 toxin, for 24 h. PAM, actively dividing and differentiated IPEC-J2 cells were subjected to T-2 toxin concentrations ranging from 0.250 to 10 ng/mL, 0.500 to 10 ng/mL and 0.500 to 100 ng/mL, respectively. To assess cytotoxicity, 150 μL of freshly prepared neutral red solution (33 μg/mL in DMEM without phenol red), preheated to 37°C, was added to each well and the plate was incubated at 37°C for an additional 2 h. The cells were then washed twice with HBSS + and the dye was released from viable cells by adding 150 μL of extracting solution ethanol/Milli-Q water/acetic acid, 50/49/1 (v/v/v) to each well. The plate was shaken for 10 min and the absorbance was determined at 540 nm using a microplate ELISA reader (Multiscan MS, Thermo Labsystems, Helsinki, Finland). The percentage of viable cells was calculated using the following formula:

$$\%\text{ cytotoxicity }=\text{ 1}00\;\times \left(\left(\text{a}-\text{b}\right)\;/\;\left(\text{c}-\text{b}\right)\right).$$

Where a = OD_540 _derived from the wells incubated with T-2 toxin, b = OD_540 _derived from blank wells, c = OD_540 _derived from untreated control wells.

#### Effect of T-2 toxin on the invasion and intracellular survival of *Salmonella *Typhimurium in porcine macrophages and intestinal epithelial cells

To examine whether the ability of *Salmonella *Typhimurium to invade and proliferate in PAM and IPEC-J2 cells was altered after exposure of these cells to T-2 toxin, invasion and intracellular survival assays were performed.

For the invasion assays, PAM and IPEC-J2 cells were seeded in 24-well plates at a density of approximately 5 × 10^5 ^and 10^5 ^cells per well, respectively. PAM were allowed to attach for 2 h and IPEC-J2 cells were allowed to grow for either 24 h or 21 days. Subsequently, PAM and actively dividing and differentiated IPEC-J2 cells were exposed to different concentrations of T-2 toxin ranging from 0.250 to 7.5 ng/mL, 0.500 to 10 ng/mL and 0.500 to 100 ng/mL, respectively. After 24 h, a gentamicin protection assay was performed as mentioned above. In short, the cells were inoculated with *Salmonella *bacteria (WT or Δ*hilA*), whether or not grown in LB medium with T-2 toxin at concentrations ranging from 0.5 to 100 ng/mL, at a MOI of 10:1. Subsequently, the cells were washed and fresh medium supplemented with 100 μg/mL gentamicin was added. After one hour, PAM and IPEC-J2 cells were washed 3 times and lysed for 10 min with 1% (v/v) Triton X-100 (Sigma-Aldrich) or 0.2% (w/v) sodium deoxycholate (Sigma-Aldrich), respectively, and 10-fold dilutions were plated on BGA plates.

To assess intracellular growth, cells were seeded and inoculated with *Salmonella *Typhimurium as described in the invasion assay, but the medium containing 100 μg/mL gentamicin was replaced after 1 h incubation with fresh medium containing 20 μg/mL gentamicin, whether or not supplemented with different concentrations of T-2 toxin as mentioned above. The number of viable bacteria was assessed 24 h after infection.

#### Effect of T-2 toxin on the translocation of *Salmonella *Typhimurium through an intestinal epithelial cell layer

To examine whether T-2 toxin affects the transepithelial passage of *Salmonella *Typhimurium through IPEC-J2 cells, a translocation assay was performed. Prior to seeding IPEC-J2 cells, Transwell^® ^polycarbonate membrane inserts with a pore size of 3.0 μm and membrane diameter of 6.5 mm (Corning Costar Corp., Cambridge, MA, USA) were coated using PureCol bovine purified collagen (Inamed Biomaterials, Fremont, California, USA). Collagen working solution was made using a 1:100 dilution of PureCol (2.9 mg/mL) in H2O. Two hundred μL of the working collagen solution was added to each transwell and was allowed to air-dry in a laminar flow hood before being exposed to UV radiation for 20 min. After coating, IPEC-J2 cells were seeded on the apical side of these inserts at a density of 2 × 10^4 ^cells/insert, cell medium was refreshed every 3 days and cells were cultured for 21 days in order to differentiate, which was determined by a preliminary experiment (Additional file 2).

After 21 days, 200 μL cell culture medium with T-2 toxin at concentrations of 0.750, 1, 2.5, 4 or 5 ng/mL was added to the apical side, while the basolateral side received 1 mL of blank culture medium. After 24 h of treatment with T-2 toxin, the Transwell^® ^inserts were washed three times with HBSS+. Then, 5 × 10^6 ^CFU of *Salmonella *Typhimurium were added to the apical compartment, suspended in IPEC-J2 medium without antibiotics, but supplemented with the respective concentrations of T-2 toxin. The basolateral compartment was filled with antibiotic-free IPEC-J2 medium. After 15, 30, 45 and 60 min at 37°C and 5% CO_2_, the number of bacteria (CFU/mL) was determined in the basolateral compartment by plating 10-fold dilutions on BGA plates. In addition, transepithelial electrical resistance (TEER) measurements were performed before and after the incubation with T-2 toxin in order to evaluate the cell barrier integrity. This was done by transferring the inserts to an insert chamber (EndOhm-6, World Precision Instruments, Sarasota, Florida, USA) and measuring the TEER via an epithelial voltohmmeter (World Precision Instruments).

### Effects of T-2 toxin on porcine host cells

In order to elucidate the underlying mechanism of T-2 toxin induced increased invasion and translocation of *Salmonella *Typhimurium in and over porcine host cells, the effects of T-2 toxin on porcine host cells were assessed.

#### Cytotoxicity of T-2 toxin towards porcine macrophages and intestinal epithelial cells

In order to determine the toxic character of T-2 toxin on porcine host cells and to determine whether it increases the toxicity of *Salmonella *Typhimurium for these porcine host cells, the cytotoxicity of T-2 toxin on uninfected PAM and IPEC cells was determined as described in the neutral red assay.

#### Effect of T-2 toxin on porcine enterocyte ultrastructure

Since the invasion assay pointed out that the T-2 toxin induced increased invasion of *Salmonella *Typhimurium was the highest in differentiated IPEC-J2 cells, transmission electron microscopy (TEM) was performed to characterize the effects of T-2 toxin on the ultrastructure of differentiated IPEC-J2 cells. The effect of 5 ng/mL T-2 toxin was investigated because this concentration significantly increases the invasion of the bacterium, without affecting the cell viability.

IPEC-J2 cells were seeded in 24-well plates at a density of approximately 10^5 ^cells per well and were allowed to grow for 21 days. Samples for TEM were collected 24 h after treatment with 5 ng/mL T-2 toxin or blank medium as a control. After treatment with T-2 toxin, the wells were washed three times with HBSS+, after which the cells were fixed in 4% formaldehyde in a 0.121 M Na-cacodylate buffer (pH 7.4) containing 1% (w/v) CaCl_2 _for 24 h. After fixation, the wells were rinsed and subsequently dehydrated by adding successively 50%, 70%, 90% and 100% ethanol to the wells. Next, the cells were embedded in LX-112 resin (Ladd Research Industries, Burlington, Vermont, USA) and cut with an ultratome (Ultracut E, Reichert Jung, Nussloch, Germany). The sections were examined under a Jeol EX II transmission electron microscope (Jeol, Tokyo, Japan) at 80 kV.

#### Effect of T-2 toxin on the protein expression of porcine enterocytes

Based on the results of the invasion assay, a comparative proteome study was conducted to reveal the effects of 5 ng/mL T-2 toxin on the protein expression of differentiated IPEC-J2 cells. We used a gel-free approach called isobaric tags for relative and absolute quantification (iTRAQ) in which four different isobaric labels are used to tag N-termini and lysine side chains of four different samples with four different isobaric reagents. Upon collision-induced dissociation during MS/MS, the isobaric tags are released, which results in four unique reporter ions that are used to quantify the proteins in the four different samples [27].

##### Sample preparation

IPEC-J2 cells were seeded in 175 cm^2 ^cell culture flasks at a density of approximately 2 × 10^6 ^cells per flask and were allowed to grow for 21 days. Subsequently, IPEC-J2 cells were washed 3 times with HBSS + and either incubated with 5 ng/mL T-2 toxin or left untreated. After 24 h, the cells were washed 3 times with HBSS + and were scraped off the bottom of the flask using a cell scraper. After washing the cells by centrifugation at 2300 × *g *for 10 min at 4°C, they were finally resuspended in 500 μL lysis buffer containing 40 mM Tris(hydoxymethyl)aminomethane hydrochloride (Tris, Sigma-Aldrich), a cocktail of protease inhibitors (PIs; Sigma-Aldrich) and phosphatase inhibitors (PPI, Sigma-Aldrich), 172.6 U/mL deoxyribonuclease I (DNase I, Invitrogen, USA), 100 mg/mL ribonuclease A (RNase A, Qiagen, Venlo, The Netherlands) and 2% (v/v) tributylphosphine (TBP, Sigma-Aldrich). The cells were sonicated (6 times 30 s), using an ultrasonic processor XL 2015 (Misonix, Farmingdale, New York, USA), followed by centrifugation at 17 968 × *g *for 10 min. The supernatant was held on ice until further use and the pellet was dissolved and sonicated (6 times 30 s), in reagent 3 of the Ready Prep Sequential extraction kit (Bio-Rad, Hercules, CA, USA). This was centrifugated at 17 968 × *g *for 10 min. Both supernatants were combined and a buffer switch to 0.01% (w/v) SDS in H_2_O was performed using a Vivaspin column (5000 molecular weight cut off Hydrosarts, Sartorius, Germany). Protein concentration was determined using the Bradford Protein Assay (Thermo Fisher Scientific, Rockford, USA) according to the manufacturer's instructions.

##### Trypsin digest and iTRAQ labeling

Digest and labeling of the samples (100 μg proteins per sample) with iTRAQ reagents was performed according to the manufacturer's guidelines (AB Sciex, Foster City, CA, USA). Individual samples of T-2 toxin treated or untreated IPEC-J2 cells were analyzed in the same run, making pairwise comparisons possible and minimizing technical variation. Each condition was run in duplicate using different labels of the four-plex labeling kit. The experiment was conducted in twofold and the labeling of the samples was as follows: run 1 (untreated IPEC-J2 cells sample 1: 114 - untreated IPEC-J2 cells sample 2: 115 - treated IPEC-J2 cells sample 1: 116 - treated IPEC-J2 cells sample 2: 117) - run 2 (untreated IPECJ-2 cells sample 3: 114 - untreated IPEC-J2 cells sample 4: 115 - treated IPEC-J2 cells sample 3: 116 - treated IPEC-J2 cells sample 4: 117). After labeling, 6 μL of a 5% (v/v) hydroxylamine solution was added to hydrolyze unreacted label and after incubation at room temperature for 5 min, the samples were pooled, dried and resuspended in 5 mM KH_2_PO_4 _(15% (v/v) acetonitrile) (pH 2.7). The combined set of samples was first purified on ICAT SCX cartridges, desalted on a C18 trap column and finally fractionated using SCX chromatography. Each fraction was analyzed by nano LC-MS/MS as described by Bijttebier et al. [28].

##### Data analysis

With no full pig protein database available, different search parameters and databases, both EST and protein, were validated to obtain maximum spectrum annotation. Best results (39% of spectra annotated above homology threshold with a 3.71% false discovery rate in the decoy database) were obtained when searching NCBI Mammalia. For quantification, data quality was validated using ROVER [29]. Based on this validation a combined approach was used to define recurrently different expression patterns. In a first approach, the four ratios that can be derived from each run (114/116, 115/117, 114/117 and 115/116) were log-transformed and a *t*-test was used to isolate protein ratios significantly different from 0 in each run. In a second approach, the two runs were merged into one file and the 114/116 and 115/117 ratios of each run were log-transformed and these ratios were multiplied (log*log). Proteins with recurrent up- or downregulation result in positive log*log protein ratios and those > 0.01 were retained and listed. Proteins that were present in both lists were considered unequivocally differentially expressed. This combined approach allows defining proteins with relatively low, but recurrent expressional differences.

### Effect of T-2 toxin on the growth, gene expression and motility of *Salmonella *Typhimurium

Not only porcine host cells, but also *Salmonella *bacteria come in contact with T-2 toxin. Therefore, it is possible that T-2 toxin affects the bacterium and by doing so, alters the pathogenesis of a *Salmonella *Typhimurium infection in pigs.

#### Effect of T-2 toxin on the gene expression of *Salmonella *Typhimurium

To test whether T-2 toxin affects the gene expression of *Salmonella *Typhimurium, a microarray analysis was performed on RNA isolated from cultures of *Salmonella *Typhimurium grown for 5 hours to a logarithmic phase in the presence or absence of 5 ng/mL of T-2 toxin.

Two OD600 units were harvested and RNA was extracted and purified using the SV Total RNA Isolation Kit (Promega Benelux bv, Leiden, The Netherlands) according to manufacturers' instructions. The quality and purity of the isolated RNA was determined using a Nanodrop spectrophotometer and Experion RNA StdSens Analysis kit (Biorad). The SALSA microarrays and protocols for RNA labeling, microarray hybridization and subsequent data acquisition have been described previously [30]. RNA (10 μg) from 3 independent biological replicates of T-2 toxin treated and untreated (control) logarithmic phase cultures was labeled with Cy5 dCTP and hybridized to SALSA microarrays with 400 ng of Cy3 dCTP labeled gDNA, as a common reference.

Genes were assessed to be statistically significantly differently expressed between the T-2 toxin treated and untreated controls by an analysis of variance test with a Benjamini and Hochberg false discovery rate of 0.05 and with a 1.5-fold change in the expression level.

The microarray data discussed in this publication are MIAME compliant and have been deposited in NCBI's Gene Expression Omnibus [31] and are accessible through GEO Series accession number GSE30925 [32].

#### Effect of T-2 toxin on the growth of *Salmonella *Typhimurium

The effect of T-2 toxin (0, 0.04, 0.31, 2.5 and 20 μg/mL) on the growth of *Salmonella *Typhimurium was examined during 24 h. For this purpose, *Salmonella *Typhimurium was grown in LB broth whether or not supplemented with T-2 toxin. The number of CFU/mL was determined at different time points (*t *= 0, 3, 6, 8 and 24 h) by titration of 10-fold dilutions of the bacterial suspensions on BGA.

#### Effect of T-2 toxin on the motility of *Salmonella *Typhimurium

One μL of an overnight culture of *Salmonella *Typhimurium was spotted in the middle of a swim plate (Difco Nutrient Broth (Becton, Dickinson and Company, Sparks, USA), 0.5% (w/v) glucose, 0.25% agar), whether or not supplemented with T-2 toxin (0, 100, 500, 1000 ng/mL). The plates were allowed to dry for 1 h at room temperature, after which they were incubated at 37°C for 16 h.

### Statistical analysis

All in vitro experiments were conducted in triplicate with 3 repeats per experiment, unless otherwise stated. All statistical analyses were performed using SPSS version 19 (SPSS Inc., Chicago, IL, USA). Normally distributed data were analyzed using unpaired Student's *t*-test or one-way ANOVA to address the significance of difference between mean values with significance set at *p *≤ 0.05. Bonferroni as post hoc test was used when equal variances were assessed. If equal variances were not assessed, the data were analyzed using Dunnett's T3 test. Not normally distributed data were analyzed using the non parametric Kruskal-Wallis analysis, followed by a Mann-Whitney *U *test.

## Results

### T-2 toxin decreases the amount of *Salmonella *Typhimurium bacteria present in the cecum contents of pigs and causes a reduction in weight gain

**Figure 1:**
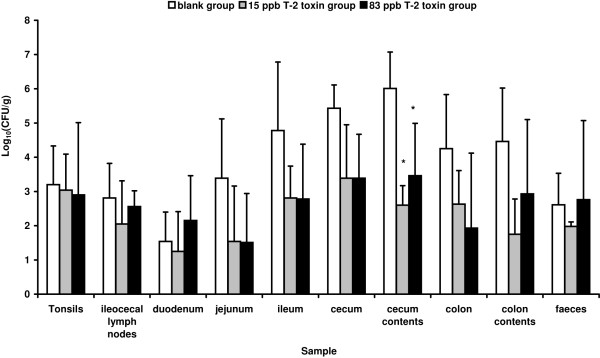
**Effect of T-2 toxin on the colonization by *Salmonella *Typhimurium in pigs**. Recovery of *Salmonella *Typhimurium bacteria from various organs and gut contents of pigs that received, during 23 days, blank feed (control group, white bars), feed contaminated with 15 μg T-2 toxin per kg feed (15 ppb group, grey bars) or feed contaminated with 83 μg T-2 toxin per kg feed (83 ppb group, black bars), respectively. Five days after inoculation with 2 × 10^7 ^CFU of *Salmonella *Typhimurium, the pigs were euthanized and the log10 value of the ratio of CFU per gram sample is given as the mean + standard deviation. Superscript (*) refers to a significant difference compared to the control group (*p *< 0.05).

The presented study shows the effects of low and relevant concentrations of T-2 toxin on the course of a *Salmonella *Typhimurium infection in pigs. Animals that received feed contaminated with 15 μg or 83 μg T-2 toxin per kg feed for 23 days had lower numbers of *Salmonella *Typhimurium per gram in their bowel contents and organs in comparison to the control group that received non-contaminated feed for 23 days. As illustrated in Figure 1, this decrease was significant in the cecum contents for both the 15 ppb and 83 ppb group (*p *= 0.001 and *p *= 0.011, respectively). A tendency to reduced colonization of the jejunum, ileum, cecum, colon and colon contents was noticed, although not significantly.

As shown in Table 1 the addition of 83 μg T-2 toxin per kg feed resulted in a significantly reduced weight gain in comparison to the control group that had a mean weight gain (%) ± standard deviation, during 18 days, of 102.1 ± 17.3 (*p *= 0.016). Pigs that were fed 15 or 83 μg T-2 toxin per kg feed, for 18 days had a mean weight gain (%) ± standard deviation of 104.4 ± 14.2 and 70.9 ± 11.3, respectively. This corresponds to a mean weight gain ± standard deviation of 0.326 ± 0.08 kg per day for the control group, 0.322 ± 0.08 kg per day for the 15 ppb group and 0.239 ± 0.04 kg per day for the 83 ppb group.

### The addition of T-2 toxin (15 μg/kg) to the feed causes a decreased expression of IL-1β

**Figure 2:**
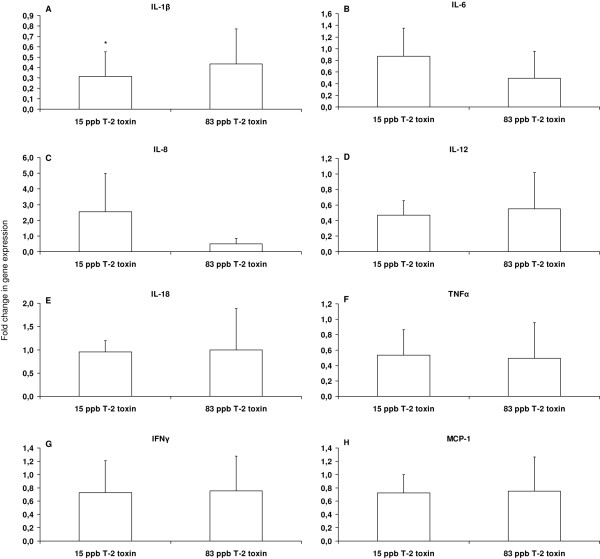
**Effect of T-2 toxin on the intestinal inflammatory response**. Fold change in cytokine gene expression of the porcine ileum of *Salmonella *Typhimurium positive pigs that received feed contaminated with 15 μg T-2 toxin per kg feed (15 ppb T-2 toxin) or feed contaminated with 83 μg T-2 toxin per kg feed (83 ppb T-2 toxin), relative to *Salmonella *Typhimurium positive pigs that received blank feed (control group), during 23 days. Five days after inoculation with 2 × 10^7 ^CFU of *Salmonella *Typhimurium, the pigs (*n *= 5) were euthanized and the cytokine gene expression levels (A: Il-1β, B: Il-6, C: Il-8, D: IL-12, E: IL-18, F: TNFα, G: IFNγ and H: MCP-1) were determined. The data represent the normalized target gene amount relative to the control group which is considered 1. The results are presented as means + standard deviation for a total of 5 pigs per test condition. Superscript (*) refers to a significant difference compared to the control group (*p *< 0.05).

The effect of a 23 day feeding period with 15 μg or 83 μg of T-2 toxin per kg feed on the intestinal mRNA expression levels of the cytokines (IL-1β, IL-6, IL-12, IL-18, IFNγ and TNFα) and chemokines (IL-8 and MCP-1) was examined 5 days post inoculation with *Salmonella *Typhimurium. The results are illustrated in Figure 2. A significant decreased fold-difference of mRNA expression ± standard deviation of pigs exposed to 15 μg T-2 toxin per kg feed was noticed compared to the *Salmonella *Typhimurium positive control pigs for IL-1β (0.31 ± 0.24) (*p *= 0.027).

### T-2 toxin is cytotoxic to porcine macrophages and intestinal epithelial cells

**Figure 3:**
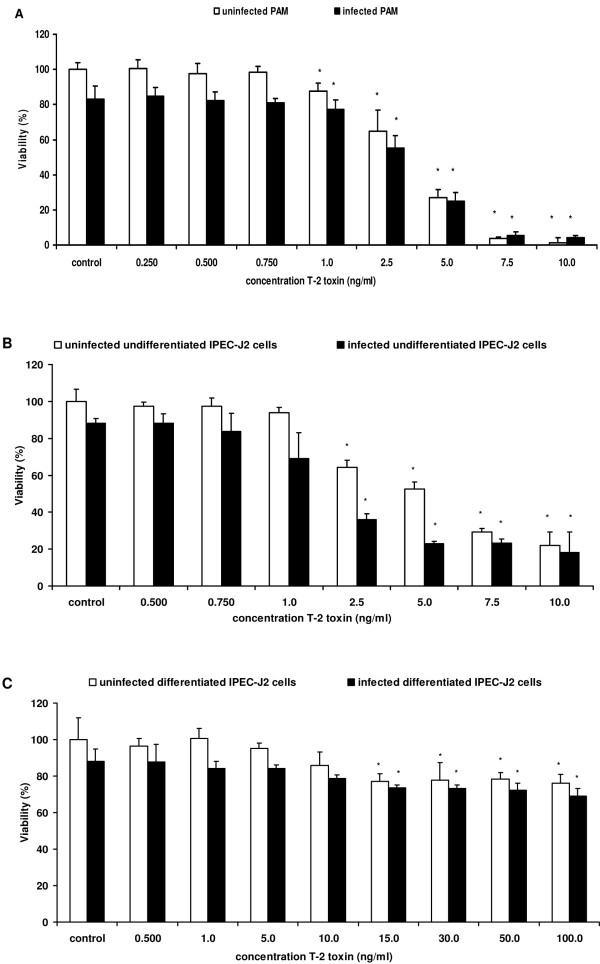
**The effect of T-2 toxin on the cell viability**. Percentage viability (%) of *Salmonella *Typhimurium infected and uninfected (**A**) PAM exposed to different concentrations of T-2 toxin (0.250-10 ng/mL), (**B**) undifferentiated IPEC-J2 cells exposed to different concentrations of T-2 toxin (0.500-10 ng/mL), (**C**) differentiated IPEC-J2 cells exposed to different concentrations of T-2 toxin (0.500-100 ng/mL). Twenty-four hours after incubation with T-2 toxin, the cytotoxic effect was determined by neutral red assay. Results represent the means of 3 independent experiments conducted in triplicate and their standard deviation. Superscript (*) refers to a significant difference compared to the control group (*p *< 0.05).

The cytotoxic effect of T-2 toxin on PAM, undifferentiated and differentiated IPEC-J2 cells as determined using the neutral red assay, is shown in Figure 3. The viability of both uninfected and infected PAM, undifferentiated and differentiated IPEC-J2 cells was significantly decreased by exposure to concentrations of T-2 toxin ≥ 1 ng/mL, ≥ 2.5 ng/mL and ≥ 15 ng/mL, respectively. IC50 values of T-2 toxin for the different cell types were determined by linear regression and are presented in Table 2.

**Table 2:** IC50 values of T-2 toxin for PAM, undifferentiated and differentiated IPEC-J2 cells, either or not infected with *Salmonella *Typhimurium.

Cell type	T-2 toxin concentration (ng/mL)
Uninfected PAM	4.3
Infected PAM	4.4
Uninfected undifferentiated IPEC-J2 cells	5.7
Infected undifferentiated IPEC-J2 cells	4.7
Uninfected differentiated IPEC-J2 cells	185
Infected differentiated IPEC-J2 cells	212.8

### Treatment of porcine macrophages and intestinal epithelial cells with T-2 toxin promotes the invasion of *Salmonella *Typhimurium

**Figure 4:**
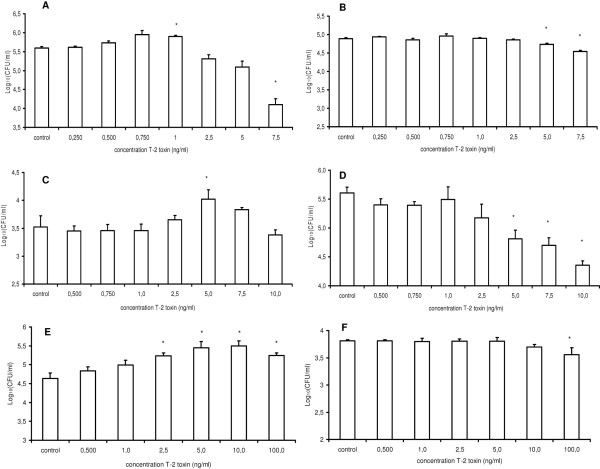
**Effect of T-2 toxin treatment of porcine cells on the invasion and intracellular proliferation of *Salmonella *Typhimurium**. The invasiveness is shown of *Salmonella *Typhimurium in (**A**) PAM, (**C**) undifferentiated and (**E**) differentiated IPEC-J2 cells whether or not exposed to different concentrations of T-2 toxin (0.250-7.5, 0.500-10 or 0.500100 ng/mL respectively). The survival of *Salmonella *Typhimurium, 24 h after invasion in (**B**) PAM, (**D**) undifferentiated and (**F**) differentiated IPEC-J2 cells whether or not exposed to different concentrations of T-2 toxin (0.250-7.5, 0.500-10 or 0.500-100 ng/mL respectively) is given. The log10 values of the number of gentamicin protected bacteria + standard deviation are given. Results are presented as a representative experiment conducted in triplicate. Superscript (*) refers to a significant difference compared to the control group (*p *< 0.05).

Altered host-pathogen interactions between *Salmonella *Typhimurium and porcine host cells could account for the reduced numbers of *Salmonella *Typhimurium present in the cecum contents. Therefore, the effect of T-2 toxin on host-pathogen interactions was investigated. The results of the invasion and intracellular survival assays of *Salmonella *Typhimurium in PAM, undifferentiated and differentiated IPEC-J2 cells with or without exposure to T-2 toxin are summarized in Figure 4.

**Figure 5:**
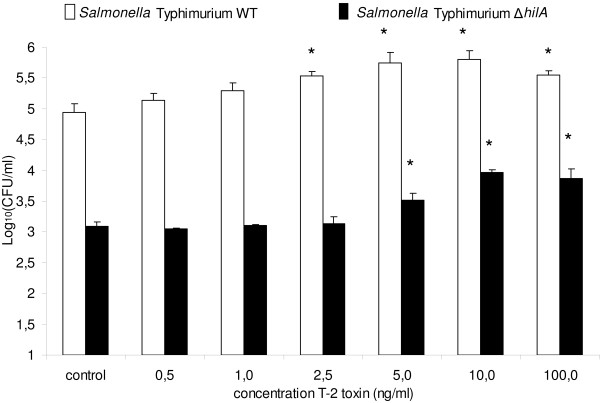
**Effect of T-2 toxin treatment of differentiated IPEC-J2 cells, on the invasion of *Salmonella *Typhimurium WT and Δ*hilA***. The invasiveness is shown of *Salmonella *Typhimurium WT (white bars) and *Salmonella *Typhimurium Δ*hilA *(black bars) in differentiated IPEC-J2 cells whether or not exposed to different concentrations of T-2 toxin (0.500-100 ng/mL). The log10 values of the number of gentamicin protected bacteria + standard deviation are given. Results are presented as a representative experiment conducted in triplicate. Superscript (*) refers to a significant difference compared to the control group (*p *< 0.05).

The invasion of *Salmonella *Typhimurium was higher in PAM, undifferentiated and differentiated IPEC-J2 cells that were treated with T-2 toxin, for 24 h, in comparison to non-treated cells. Exposure of PAM, undifferentiated and differentiated IPEC-J2 cells to T-2 toxin concentrations of 1, 5 and ≥ 2.5 ng/mL, respectively, led to a significant increase in the number of intracellular *Salmonella *Typhimurium bacteria. Due to the toxicity of T-2 toxin, exposure of PAM to T-2 toxin concentrations ≥ 7.5 ng/mL, resulted in a significant decrease in the number of intracellular bacteria. As shown in Figure 5, similar results were obtained using the deletion mutant Δ*hilA*, where a significant increased invasion was seen at T-2 toxin concentrations > 5 ng/mL.

A 24 h treatment of *Salmonella *infected PAM, undifferentiated and differentiated IPEC-J2 cells with non-cytotoxic concentrations of T-2 toxin, did not affect the intracellular proliferation of *Salmonella *Typhimurium in these cells. However, treatment with toxic concentrations of T-2 toxin resulted in a significantly decreased survival of *Salmonella *Typhimurium in PAM and undifferentiated IPEC-J2 cells at T-2 toxin concentrations ≥ 5 ng/mL T-2 toxin and in differentiated IPEC-J2 cells at concentrations ≥ 100 ng/mL T-2 toxin.

### T-2 toxin promotes the transepithelial passage of *Salmonella *Typhimurium through the intestinal epithelium

**Figure 6:**
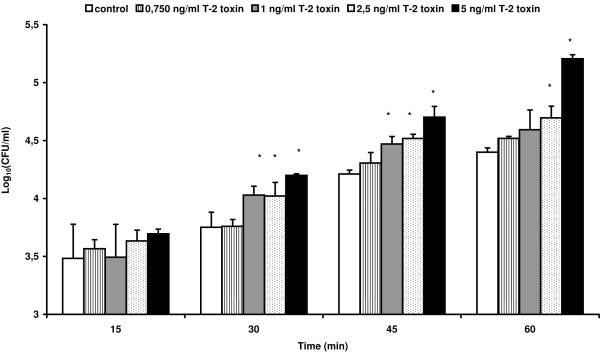
**The influence of T-2 toxin treatment of an on IPEC-J2 monolayer on the transepithelial passage of *Salmonella *Typhimurium**. IPEC-J2 cells seeded onto inserts for 21 days until differentiation were either exposed to blank medium or treated with different concentrations of T-2 toxin (0.750, 1, 2.5 or 5 ng/mL) for 24 h, prior to measuring the transepithelial passage of *Salmonella *Typhimurium. The translocation of the bacteria was measured 15, 30, 45 and 60 min after inoculation. Results are presented as a representative experiment conducted in triplicate. Superscript (*) refers to a significantly higher translocation of the bacteria compared to the unexposed control wells (*p *< 0.05).

The passage of *Salmonella *Typhimurium through 21-days-old IPEC-J2 cells treated for 24 h with non-cytotoxic concentrations of T-2 toxin varying from 0.750 to 5 ng/mL is shown in Figure 6. Already after 30 min, treatment of the IPEC-J2 cell monolayer with T-2 toxin concentrations ≥ 1 ng/mL resulted in a significant increase in the number of translocated bacteria in comparison to non-treated IPEC-J2 cells. Exposure to concentrations of T-2 toxin varying from 0.750 to 5 ng/mL, for 24 h, did not lead to a decrease in TEER (Additional file 3) indicating no loss of integrity of the epithelial monolayer and suggesting an increased transcellular passage of the bacteria.

### T-2 toxin affects the protein expression of differentiated IPEC-J2 cells at a concentration that does not cause morphological changes

In order to elucidate the possible mechanism of the T-2 toxin increased invasion in differentiated IPEC-J2 cells, iTRAQ analysis was performed on T-2 toxin treated porcine cells. Based on the invasion assay results, we opted to investigate the effect of T-2 toxin at a concentration of 5 ng/mL on differentiated IPEC-J2 cells. Peptides from trypsin digested proteins were labeled with isobaric mass tag labels and analyzed by 2-D LC-MS/MS. Collision-induced dissociation results in the release of these isobaric tags, which allows relative quantification of the peptides. A broad comparison between 5 ng/mL T-2 toxin treated and untreated differentiated IPEC-J2 cells, resulted in the identification of 21 proteins with relatively low, but recurrent expressional differences, as shown in Table 3. Eight of these proteins showed higher levels in untreated IPEC-J2 cells, whereas 13 of them were more abundant in T-2 toxin treated IPEC-J2 cells.

**Table 3:** Differential protein expression of differentiated IPEC-J2 cells after exposure to T-2 toxin.

Protein name*	Function*	Protein ratio treated/untreated IPEC-J2 cells on the *T*-test approach	Protein ratio treated/untreated IPEC-J2 cells on the log*log approach
Cytochrome c oxidase subunit VIIc {N-terminal}	This protein is one of the nuclear-coded polypeptide chains of cytochrome c oxidase, the terminal oxidase in mitochondrial electron transport.	0.6	0.6
Microsomal glutathione S-transferase 3	Functions as a glutathione peroxidase.	0.6	0.6
PREDICTED: similar to Keratin, type I cytoskeletal 18 (Cytokeratin 18)	When phosphorylated, plays a role in filament reorganization.	0.7	0.7
Myristoylated alanine-rich C-kinase substrate	Myristoylated alanine-rich C-kinase substrate is a filamentous (F) actin cross-linking protein.	0.7	0.7
Annexin A4	Calcium/phospholipid-binding protein which promotes membrane fusion and is involved in exocytosis.	0.7	0.7
Chain A, Bovine Mitochondrial F1-Atpase Complexed With Aurovertin B	Mitochondrial membrane ATP synthase (F1F0 ATP synthase or Complex V) produces ATP from ADP in the presence of a proton gradient across the membrane.	0.7	0.8
Protein S100-A16	Calcium-binding protein. Binds one calcium ion per monomer.	0.8	0.8
Putative beta-actin	Actins are highly conserved proteins that are involved in various types of cell motility and are ubiquitously expressed in all eukaryotic cells.	0.8	0.7
Cysteine and glycine-rich protein 1 isoform 1	Encodes a member of the cysteine-rich protein (CSRP) family that includes a group of LIM domain proteins, which may be involved in regulatory processes important for development and cellular differentiation.	1.2	1.2
Heat shock protein 60	Implicated in mitochondrial protein import and macromolecular assembly.	1.2	1.2
PREDICTED: similar to nucleolin-related protein isoform 3	Plays a role in different steps in ribosome biogenesis.	1.2	1.2
Heterogeneous nuclear ribonucleoprotein F	Component of the heterogeneous nuclear ribonucleoprotein (hnRNP) complexes which provide the substrate for the processing events that pre-mRNAs undergo before becoming functional, translatable mRNAs in the cytoplasm.	1.2	1.2
Heat shock protein 10	Essential for mitochondrial protein biogenesis, together with chaperonin 60.	1.2	1.2
Thymosin beta-10	Binds to and sequesters actin monomers (G actin) and therefore inhibits actin polymerization.	1.2	1.2
Thioredoxin-related transmembrane protein 1	May participate in various redox reactions.	1.3	1.3
Glutathione S-transferase P	Conjugation of reduced glutathione to a wide number of exogenous and endogenous hydrophobic electrophiles.	1.3	1.3
14-3-3 protein sigma	Adapter protein implicated in the regulation of a large spectrum of both general and specialized signalling pathway. of G2/M progression.	1.3	1.3
Elongation factor 1-beta	Elongation factor 1-beta and Elongation factor 1-delta stimulate the exchange of GDP bound to Elongation factor 1-alpha to GTP.	1.3	1.3
Profilin	Binds to actin and affects the structure of the cytoskeleton.	1.5	1.5
Cyclophilin A or Peptidyl-prolyl cis-trans isomerase A	Peptidyl-prolyl isomerase accelerates the folding of proteins.	1.6	1.6
Branched-chain-amino-acid aminotransferase, cytosolic	Catalyzes the first reaction in the catabolism of the essential branched chain amino acids leucine, isoleucine, and valine.	1.6	1.6

Differentially expressed proteins identified in T-2 toxin (5 ng/mL) treated IPEC-J2 cells in comparison to untreated IPEC-J2 cells by use of iTRAQ analysis coupled to 2-D LC-MS/MS. Superscript (*) refers to the protein description according to the UniProtKB/Swiss-Prot protein sequence database [33]

**Figure 7:**
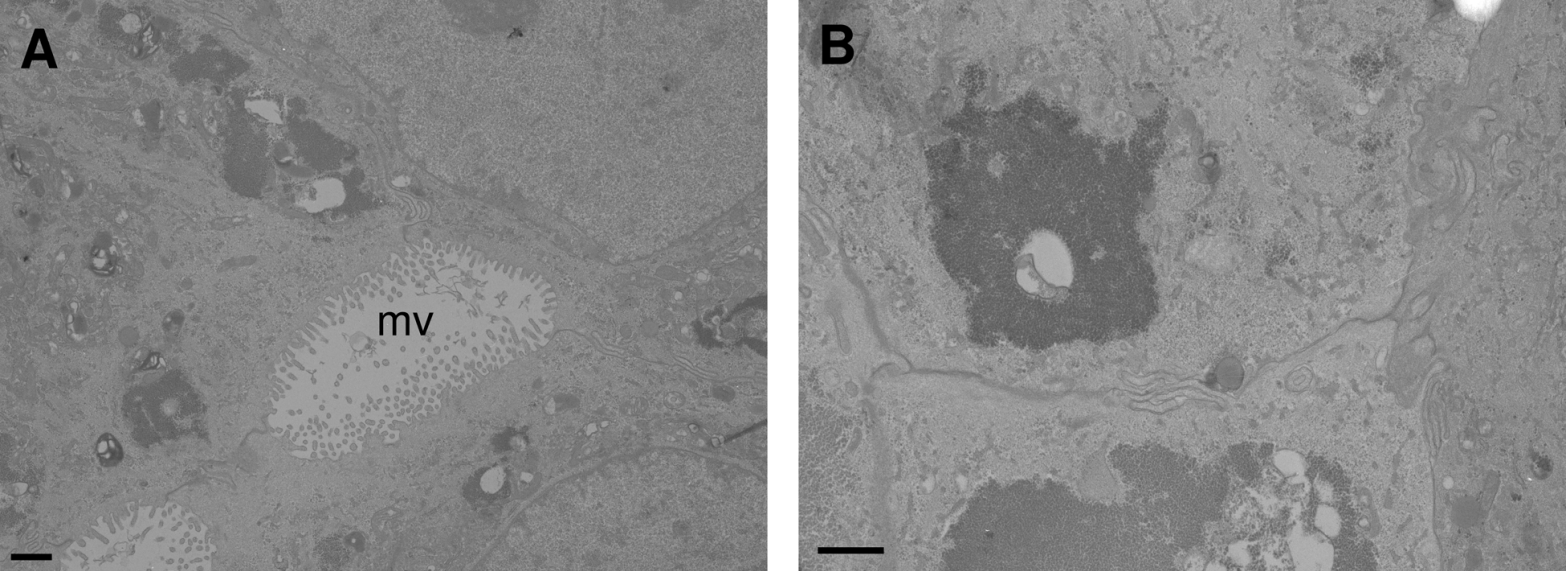
**The effect of T-2 toxin on the morphology of differentiated IPEC-J2 cells**. Transmission electron micrographs of differentiated IPEC-J2 cells fixed 24 h after exposure to (**A**) control medium or (**B**) 5 ng/mL T-2 toxin. These pictures serve as a representative for a confluent monolayer of IPEC-J2 cells and no differences were seen on the ultrastructure of T-2 toxin (5 ng/mL) treated IPEC-J2 cells in comparison to untreated cells. Scale bar = 1 μM; mv = microvilli.

Proteomic analysis established a T-2 toxin induced upregulation of proteins involved in ribosome biogenesis [34], protein synthesis and folding [35,36], or c-Jun N-Terminal kinase signalling [37], respectively predicted nucleolin-related protein isoform 3, elongation factor 1-beta, peptidyl-prolyl cis-trans isomerase A and glutathione S-transferase P. Furthermore, T-2 toxin increased the expression of pre-mRNA splicing factor heterogeneous nuclear ribonucleoprotein F, 14-3-3 sigma, branched-chain-amino-acid aminotransferase, heat shock protein 60, heat shock protein 10 and thioredoxin-related transmembrane protein 1, highlighting the toxic character of 5 ng/mL T-2 toxin. In contrast, T-2 toxin caused a decreased expression of proteins involved in membrane functions, mitochondrial proteins and endoplasmatic reticulum (ER) related proteins, namely annexin A4, cytochrome c oxidase subunit VIIc, chain A mitochondrial F1-ATPase complexed with aurovertin B, S-transferase 3 and S100-A16. These are involved in membrane bilayer function [38], mitochondrial electron transport [39], adenosine triphosphate (ATP) production [40], the cellular defense against oxygen-free radicals [41] or Ca^2+ ^homeostasis, cell proliferation, migration, differentiation, apoptosis and transcription [42,43], respectively. Moreover, T-2 toxin affects the expression of cytoskeleton associated proteins. It causes a decreased expression of cytokeratin 18, myristoylated alanine-rich C-kinase substrate and putative beta-actin and an increased expression of thymosin beta-10, cysteine and glycine-rich protein 1 isoform 1 and profiling. Generally, these data showed that even a low concentration of 5 ng/mL T-2 toxin damages the porcine enterocyte and affects cytoskeletal proteins. TEM pointed out that these changes in protein expression are not correlated with morphological changes (Figure 7).

### T-2 toxin does not affect the growth, but decreases the motility and invasiveness of *Salmonella *Typhimurium

**Figure 8:**
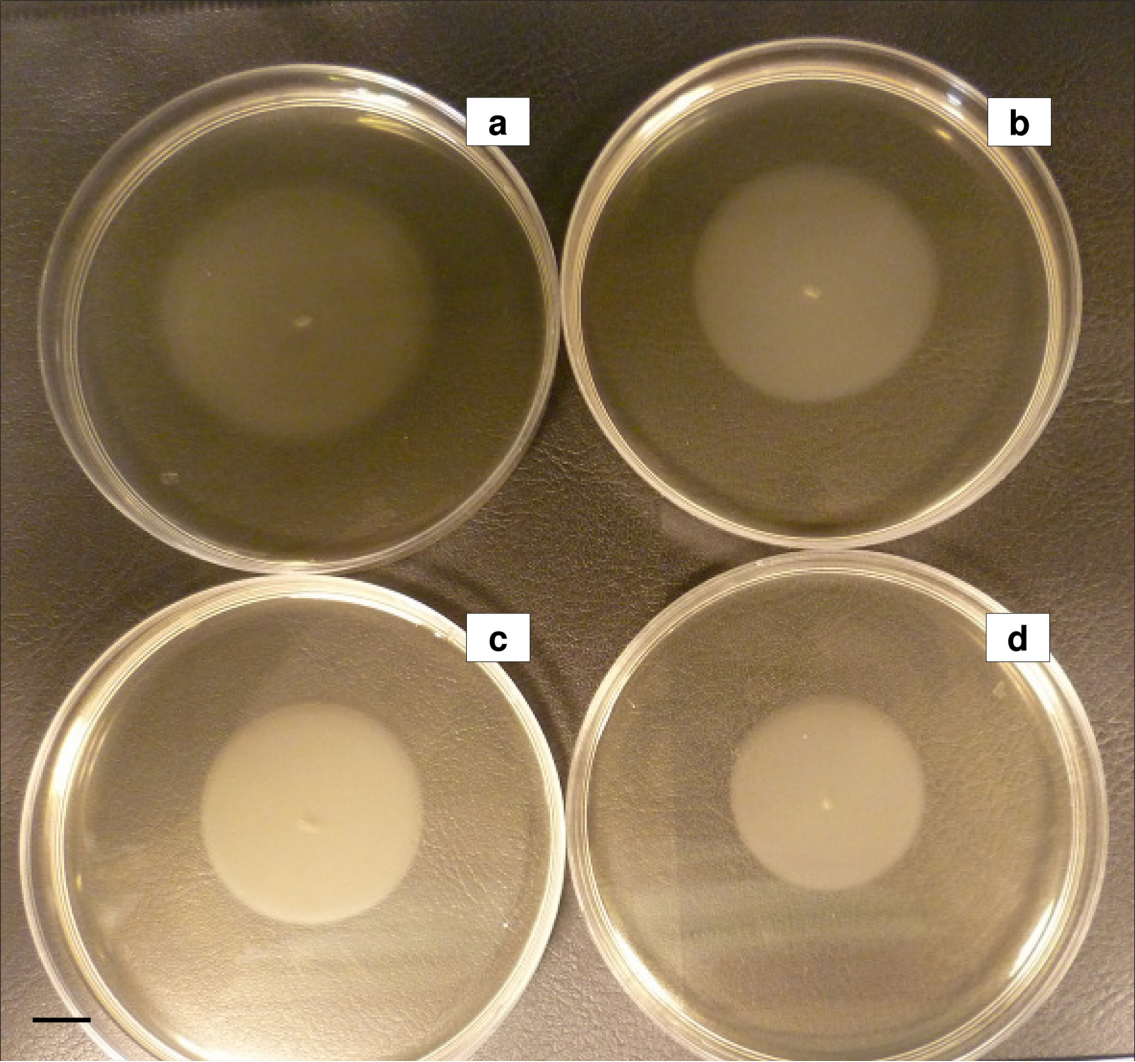
**Effect of T-2 toxin on the swarming capacity of *Salmonella *Typhimurium**. Swarming capacity of *Salmonella *Typhimurium after overnight incubation at 37°C on semi-solid agar plates supplemented with (**a**) 0 ng/mL T-2 toxin, (**b**) 100 ng/mL T-2 toxin, (**c**) 500 ng/mL T-2 toxin, or (**d**) 1000 ng/mL T-2 toxin. The diameter of the circle is a measure for the motility of the bacteria. Scale bar = 1 cm.

**Figure 9:**
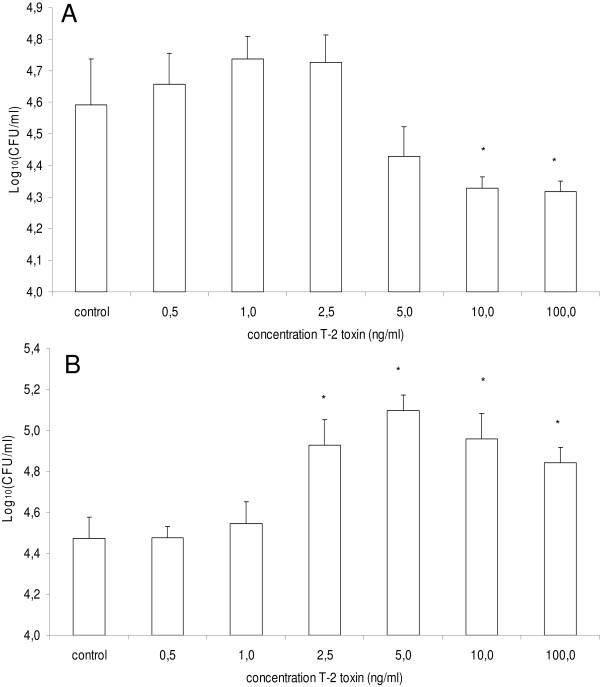
**The influence of T-2 toxin treatment of differentiated IPEC-J2 cells and/or *Salmonella *Typhimurium bacteria, on the invasion in these cells**. The invasiveness is shown of *Salmonella *Typhimurium bacteria grown for 5 h in LB medium with T-2 toxin (0.500-100 ng/mL), in (**A**) untreated differentiated IPEC-J2 cells and (**B**) T-2 toxin (0.500-100 ng/mL) treated differentiated IPEC-J2 cells, for 24 h. The log10 values of the number of gentamicin protected bacteria + standard deviation are given. Results are presented as a representative experiment conducted in triplicate. Superscript (*) refers to a significant difference compared to the control group (*p *< 0.05).

Preliminary experiments showed that T-2 toxin up to 20 μg/mL had no observable effect on the growth of *Salmonella *Typhimurium (Additional file 4). In order to look further for any possible effects of T-2 toxin on *Salmonella *Typhimurium, a microarray study was carried out with RNA isolated from logarithmic phase cultures grown for 5 h in the presence or absence of 5 ng/mL T-2 toxin. It was found that expression of 262 genes was repressed and 352 genes induced following exposure to T-2 toxin (Additional file 5 and Additional file 6). In general, exposure of *Salmonella *Typhimurium to T-2 toxin resulted in a small but significant reduction in the expression of key metabolic genes including 8 glycolytic genes, and genes encoding cytochrome o and d terminal oxidases, succinate dehydrogenase, NADH dehydrogenase and ATP synthase. Similarly, T-2 toxin exposure resulted in reduced expression of genes encoding both 30s and 50s ribosomal proteins. In addition, it was noted that expression of 5 flagella biosynthesis genes was reduced as was expression of 16 of the *Salmonella *Pathogenicity Island 1 (SPI-1) genes. Consistent with the observed reduction in flagella gene expression, motility of *Salmonella *Typhimurium on swarm plates was found to be reduced by T-2 toxin in a dose dependent manner, however at concentrations of T-2 toxin ≥ 100 ng/mL (Figure 8). Furthermore, in line with the reduced expression of the *Salmonella *SPI-1 genes, concentrations of T-2 toxin ≥ 10 ng/mL significantly decreased the invasion capacity of *Salmonella *Typhimurium in differentiated IPEC-J2 cells (Figure 9a). However, if both *Salmonella *bacteria and differentiated IPEC-J2 cells are exposed to T-2 toxin (≥ 2.5 ng/mL), increased invasion in differentiated IPEC-J2 cells was observed (Figure 9b).

Of the genes found to be upregulated following T-2 toxin exposure many (36) were related to cell-envelope and outer membrane biogenesis suggesting that the toxin may cause membrane or cell wall damage. Expression of 61 genes involved in signal transduction and transcription was increased, suggesting the bacteria were undergoing a global stress response to deal with the toxic insult. Consistent with this, both the *emrAB *and *bcr *multidrug efflux systems and the *marAB *multi-antibiotic efflux system were upregulated as were several other detoxification systems and the *yehYXW *proline/glycine betaine transport systems involved in osmoprotection. Overall the transcriptomic data reveals a bacterium under stress, up-regulating stress response systems and downregulating its metabolic functions.

## Discussion

The ingestion of T-2 toxin contaminated feed by pigs resulted in a significant reduction of weight gain, after only 18 days, compared to control pigs that received blank feed (Table 1). To our knowledge, this is the first time such an effect has been reported due to a low concentration of T-2 toxin. Since contamination of human foodstuff with T-2 toxin is an emerging issue and concentrations up to 1810 μg T-2 toxin per kg wheat have been reported in Germany [9], it is feasible that T-2 toxin may also affect human metabolism. Different studies describe that, at high doses, T-2 toxin affects the intestinal absorption of nutrients and reduces the daily feed intake, resulting in a reduced body weight gain [44-46]. However, due to the housing conditions of the animals, we were not able to record the daily feed intake of the animals. Therefore, we cannot conclude whether the reduced weight gain of the pigs was the result of a decreased daily feed intake.

iTRAQ analysis showed that even an extreme low concentration of 5 ng/mL T-2 toxin affects protein expression in differentiated IPEC-J2 cells compared to untreated cells (Table 3). The main mechanism by which T-2 toxin causes its toxic effects is through inhibition of protein synthesis, leading to a ribotoxic stress response. This activates c-Jun N-terminal kinase (JNK)/p38 MAPKs and as a consequence modulates numerous physiological processes including cellular homeostasis, cell growth, differentiation and apoptosis [47]. Proteomic analysis showed an upregulation of proteins involved in ribosome biogenesis, protein synthesis, protein folding and c-Jun N-Terminal kinase signalling. The increased expression of these proteins could be a rescue mechanism, highlighting that even a low concentration of 5 ng/mL T-2 toxin leads to a ribotoxic stress response in differentiated IPEC-J2 cells. The toxic character of T-2 toxin was also shown by the upregulation of heat shock proteins, pre-mRNA splicing factor heterogeneous nuclear ribonucleoprotein F, which could be a mechanism to increase mRNA stability [48], and 14-3-3 sigma. The protein 14-3-3 sigma is a p53-regulated inhibitor of G2/M progression [49] and its upregulation might emphasize the DNA damage caused by T-2 toxin [50]. Overall, these iTRAQ data may indicate that T-2 toxin damages the porcine enterocyte, and by doing so, harms the absorption of nutrients with a reduced weight gain as result.

In contrast to other *Fusarium *mycotoxins, there is no guidance value set by the European Commission for the amount of T-2 toxin in complementary and complete feed for pigs. As shown by a neutral red assay, T-2 toxin affects cell viability at very low concentrations (Figure 3). The in vitro viability of porcine macrophages, undifferentiated and differentiated porcine intestinal epithelial cells was significantly decreased at concentrations ≥ 1 ng/mL, ≥ 2.5 ng/mL and ≥ 15 ng/mL, respectively. Taking into account that such low concentrations negatively affect cell viability in vitro, and that these concentrations are relevant in practice [9,23], it is of utmost importance that maximum levels are set for this mycotoxin as well.

Ingestion of low and relevant concentrations of T-2 toxin results in reduced numbers of *Salmonella *Typhimurium bacteria in the cecum contents of pigs, and a tendency to a reduced colonization of the jejunum, ileum, cecum, colon and colon contents (Figure 1). With T-2 toxin and *Salmonella *Typhimurium being major problems in swine industry and with salmonellosis being one of the most important zoonotic bacterial diseases in both developed and developing countries, we aimed at evaluating the effect of T-2 toxin on the pathogenesis of a *Salmonella *Typhimurium infection in pigs. Until now, conflicting results have been published concerning the effect of mycotoxins on the susceptibility to intestinal infections and still little is known about the effects of low concentrations of these mycotoxins [16-18,51-53]. According to Ziprin and McMurray, T-2 toxin did not affect the course of salmonellosis in mice [51]. In the present study, we provide evidence that these data cannot be extrapolated to a pig host. Since the porcine intestine shows physiological, anatomical and pathological similarities to the human gut [54], it is not unlikely that T-2 toxin similarly affects the pathogenesis of a *Salmonella *infection in the human host as in the pig host.

The ingestion of 15 μg T-2 toxin per kg feed resulted in a significant decreased expression of IL-1β (Figure 2). Once *Salmonella *has invaded the intestinal epithelium, the innate immune system is triggered and the porcine gut will react with the production of several cytokines [55]. Both *Salmonella *and mycotoxins affect the innate immune system. Zhou et al. found that deoxynivalenol (DON) increases the expression of TGF-β and IFN-γ in the small intestine of mice [56]. Recently, Kruber et al. established that T-2 toxin strongly induces IL-8 production in a Caco-2 intestinal epithelial cell line [57]. According to Vandenbroucke et al., DON and *Salmonella *Typhimurium synergistically potentiate intestinal inflammation in an ileal loop model of pigs [24]. As our control group is *Salmonella *positive, we cannot conclude whether the decreased expression of IL-1β in the T-2 toxin treated pigs is caused by the effects of T-2 toxin on the innate immune system, the reduced numbers of *Salmonella *Typhimurium in the gut, or a combination of both. Furthermore, by the use of ELISA analysis, Maresca et al. showed that DON caused a biphasic effect on IL-8 secretion by Caco-2 cells [58]. They also pointed out that this biphasic effect was not observed at mRNA level, where a dose-dependent increase in IL-8 mRNA was noticed [58]. These data implicate that in order to obtain results about the secretion of IL-1β, ELISA analysis on the ileum should be performed.

In order to elucidate how T-2 toxin causes reduced numbers of *Salmonella *Typhimurium bacteria in the cecum contents of pigs, and a tendency to a reduced colonization of the jejunum, ileum, cecum, colon and colon contents, we investigated the effects of T-2 toxin on the interactions of *Salmonella *Typhimurium with porcine macrophages and intestinal epithelial cells, two cell types that play an important role in the pathogenesis of a *Salmonella *infection. In vitro treatment of the host cells with T-2 toxin rendered them more susceptible to invasion, in a SPI-1 independent manner, and increased the transepithelial passage of the bacterium (Figures 4 and 6). This is in accordance with Vandenbroucke et al. who showed that DON promotes the invasion and translocation of *Salmonella *Typhimurium over porcine host cells, by a mechanism that is not SPI-1 dependent [24,59]. The results obtained by Maresca et al. also confirm our results since they pointed out that DON concentrations that do not compromise the barrier function, significantly increase the passage of non-invasive *Escherichia coli *bacteria through Caco-2 inserts [58]. As reviewed by Maresca and Fantini [60] such increase in bacterial passage through intestinal epithelial cells could be involved in inducing inflammatory bowel diseases. Extrapolating these results to the in vivo situation, one would expect an increased colonization by *Salmonella *in pigs. However, we showed that ingestion of low and relevant concentrations of T-2 toxin resulted in a significantly decreased amount of *Salmonella *Typhimurium bacteria in the cecum contents and in a tendency to reduced colonization of the jejunum, ileum, cecum, colon and colon contents. In vitro, T-2 toxin decreased the intracellular survival of *Salmonella *Typhimurium in PAM, undifferentiated IPEC-J2 cells and differentiated IPEC-J2 cells (Figure 4) at concentrations which significantly reduced the cell viability (Figure 3). Possibly this reduced survival plays an important role in the in vivo outcome. However, whether this reduced survival is due to a decrease in viable cells, a diminished replication capacity of the bacterium or a combination of both, is unknown.

Invasion of *Salmonella *in nonphagocytic cells involves a series of cytoskeletal changes, characterized by actin polymerization and the formation of membrane ruffles. These cytoskeletal changes are important for the uptake and the cytoplasmic transport of the bacterium, as well as for the establishment and the stability of the bacterial replicative niche, also called *Salmonella *containing vacuole (SCV) [59]. By the use of iTRAQ analysis, we demonstrated that 5 ng/mL T-2 toxin induces alterations in the expression of proteins that are involved in the cytoskeleton formation of differentiated IPEC-J2 cells. T-2 toxin causes a decreased expression of cytokeratin 18, a member of the intermediate filament network that provides support and integrity to the cytoskeleton [61], of myristoylated alanine-rich C-kinase substrate, a filamentous actin crosslinking protein [62] and of putative beta-actin, which is a major component of the cytoskeleton. Furthermore, T-2 toxin causes an increased expression of thymosin beta-10, an actin-sequestering protein involved in cytoskeleton organization and biogenesis [63], of cysteine and glycine-rich protein 1 isoform 1, a regulator for actin filament bundling [64] and of profilin, an actin-binding protein that can sequester G-actin or actively participate in filament growth [65]. According to Vandenbroucke et al., low concentrations of DON can modulate the cytoskeleton of macrophages resulting in an enhanced uptake of *Salmonella *Typhimurium in porcine macrophages [59]. The observed changes in protein expression are not sufficient to induce morphological changes, as assessed with TEM (Figure 7). However, the T-2 toxin induced altered expression of cytoskeleton associated proteins could influence the interactions between IPEC-J2 cells and *Salmonella*. Thus T-2 toxin and *Salmonella *Typhimurium appear to act synergistically, inducing cytoskeleton reorganizations which increase the invasion of the bacterium.

We also examined the effects of T-2 toxin on *Salmonella *Typhimurium gene expression. Microarray analysis revealed that T-2 toxin caused a general downregulation of *Salmonella *Typhimurium metabolism (Additional file 5) and notably of ribosome synthesis. To our knowledge, this is the first time it has been shown that T-2 toxin affects ribosomal gene expression in both eukaryotic [66] and prokaryotic cells. Microarray analysis also showed that T-2 toxin causes a downregulation of flagella gene expression (Additional file 5) and consequently resulted in decreased motility of *Salmonella *Typhimurium (Figure 8). Motility of *Salmonella *increases the probability that the bacterium will reach suitable sites for invasion and successful infections [67]. Transcriptomic analysis furthermore demonstrated that exposure to T-2 toxin results in reduced expression of many SPI-1 genes. According to Boyen et al., SPI-1 plays a crucial role in the invasion and colonization of the porcine gut and in the induction of influx of neutrophils [68]. Shah et al. indicated that the pathogenicity of *Salmonella *Enteritidis isolates is associated with both motility and secretion of the type III secretion system (TTSS) effector proteins [67]. Isolates with low invasiveness had impaired motility and impaired secretion of FlgK, FljB and FlfL or TTSS secreted SipA and SipD. Therefore, a T-2 toxin induced downregulation of SPI-1 and motility genes and a reduced motility may lead to a reduced colonization by the bacterium in pigs.

In conclusion, we showed that the presence of low and in practice relevant concentrations of T-2 toxin in the feed causes a decrease in the amount of *Salmonella *Typhimurium bacteria present in the cecum contents of pigs, and a tendency to a reduced colonization of the jejunum, ileum, cecum, colon and colon contents. In vitro, T-2 toxin causes an increased invasion and transepithelial passage of the bacterium in and through T-2 toxin treated porcine cells, in a SPI-1 independent manner. However, T-2 toxin significantly reduces the SPI-1 gene expression, invasiveness and motility of the bacterium. Therefore, in vivo, the effect of T-2 toxin on the bacterium is probably more pronounced than the host cell-mediated effect.

## Competing interests

Sources of financial support have been acknowledged and the authors declare that they have no competing interests.

## Authors' contributions

EV, AT and NS performed the microarray analysis. EV and KD carried out the TEM analysis. EV, MD and DD performed the iTRAQ analysis. EV, AVP, VV, JG and BL executed the animal experiments. The T-2 toxin contaminated feed was prepared by EV, VV and ME and the concentration of T-2 toxin in the feed was determined by SDS. EV, FB, FH, PDB, SC and FP conceived the study, participated in its design and coordination. EV, SC and FP co-drafted the manuscript. VV and MD share second authorship, SC and FP share senior authorship. All authors read and approved the final manuscript.

## Supplementary Material

Additional file 1 **Composition of the blank piglet feed used in the in vivo assay**. Click here for file

Additional file 2 **The progression of TEER values of IPEC-J2 cells, seeded at a density of 2 × 10^4 ^cells, on collagen coated Transwell^® ^polycarbonate membrane inserts (pore size = 3.0 μm and membrane diameter = 6.5 mm)**. (TIFF 78 kb). Click here for file

Additional file 3 **TEER values of IPEC-J2 cells, 21 days after seeding them at density of 2 × 10^4 ^cells, on collagen coated Transwell^® ^polycarbonate membrane inserts**. After 21 days, the cells were exposed to different concentrations of T-2 toxin ranging from 0 to 5 ng/mL, during 24 h. Click here for file

Additional file 4 **Effect of T-2 toxin on the growth of *Salmonella *Typhimurium**. The log10 values of the CFU/mL + standard deviation are given at different time points (*t *= 0, 2.5, 5, 7.5, 24 h). *Salmonella *Typhimurium growth was examined in LB medium, whether or not supplemented with T-2 toxin (0.04-20 μg/mL). Results are presented as a representative experiment conducted in triplicate. Click here for file

Additional file 5 **Gene expression comparison between a logarithmic phase culture of *Salmonella *Typhimurium whether or not exposed to T-2 toxin**. Microarray data of a logarithmic phase culture of *Salmonella *Typhimurium grown in presence or absence of 5 ng/mL T-2 toxin, showing genes differentially downregulated, by ≥ 1.5 fold with *p *≤ 0.05. Click here for file

Additional file 6 **Gene expression comparison between a logarithmic phase culture of *Salmonella *Typhimurium whether or not exposed to T-2 toxin**. Microarray data of a logarithmic phase culture of *Salmonella *Typhimurium grown in presence or absence of 5 ng/mL T-2 toxin, showing genes differentially upregulated, by ≥ 1.5 fold with *p *≤ 0.05. Click here for file
