# Supplementary material for: T-2 toxin induced Salmonella Typhimurium intoxication results in decreased Salmonella numbers in the cecum contents of pigs, despite marked effects on Salmonella-host cell interactions
Source: Vet Res. 2012 Mar 22;43(1):22. doi: 10.1186/1297-9716-43-22 (PMC3362764; doi:10.1186/1297-9716-43-22)
Supplement: Additional file 1 — Composition of the blank piglet feed used in the in vivo assay. [file 1297-9716-43-22-S1.DOC]

| **Composition piglet feed** |  |
| --- | --- |
| Crude protein | 17,70% |
| Crude fat | 5,90% |
| Crude ash | 5,30% |
| Crude fiber |  |
| Phosphorus | 56,00% |
| Lysine | 1,30% |
|  |  |
| **Additives** |  |
| Vitamin A | 18500 IU/kg |
| Vitamin D3 | 2000 IU/kg |
| Vitamin E | 100 mg/kg |
| Copper(II) sulfate pentahydrate | 160 mg/kg |
| Ethoxyquin |  |
| 3-phytaseE.C.3,1,3,8(E1600) | 500 FTU/kg |
|  |  |
| **Feedstuff** |  |
| Barley | 30% |
| Wheat | 18% |
| Toasted soybeans | 14% |
| Maize | 13% |
| Soya meal | 7% |
| Wheat gluten | 4% |
| Monocalcium phosphate | 0,50% |
| Natriumchloride | 0,40% |
| Palm oil | 0,30% |
