# Supplementary figures and images for: T-2 toxin induced Salmonella Typhimurium intoxication results in decreased Salmonella numbers in the cecum contents of pigs, despite marked effects on Salmonella-host cell interactions
Source: Vet Res. 2012 Mar 22;43(1):22. doi: 10.1186/1297-9716-43-22 (PMC3362764; doi:10.1186/1297-9716-43-22)

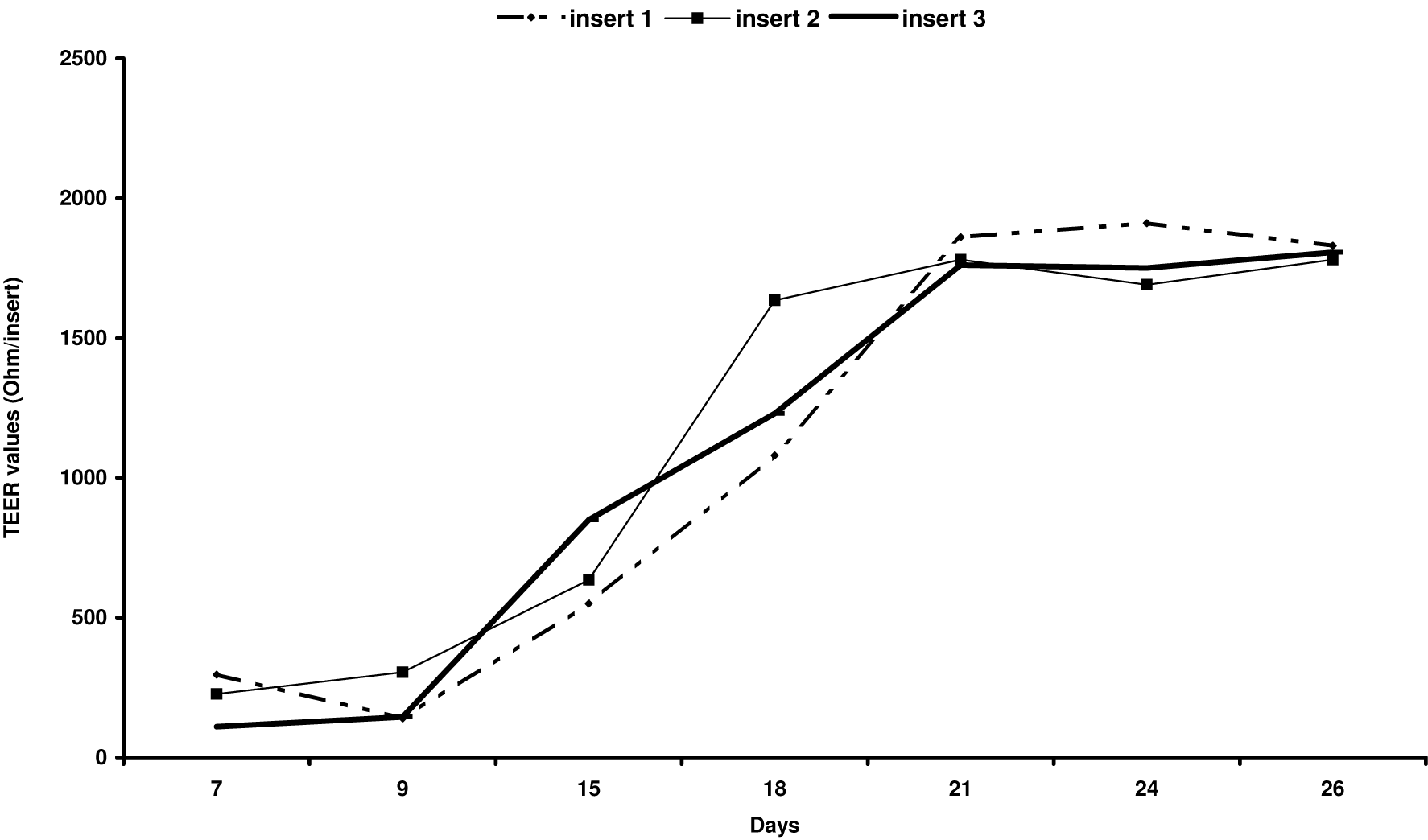

Supplement: Additional file 2 — The progression of TEER values of IPEC-J2 cells, seeded at a density of 2 × 10^4^ cells, on collagen coated Transwell® polycarbonate membrane inserts (pore size = 3.0 μm and membrane diameter = 6.5 mm). (TIFF 78 kb). [file 1297-9716-43-22-S2.TIFF]

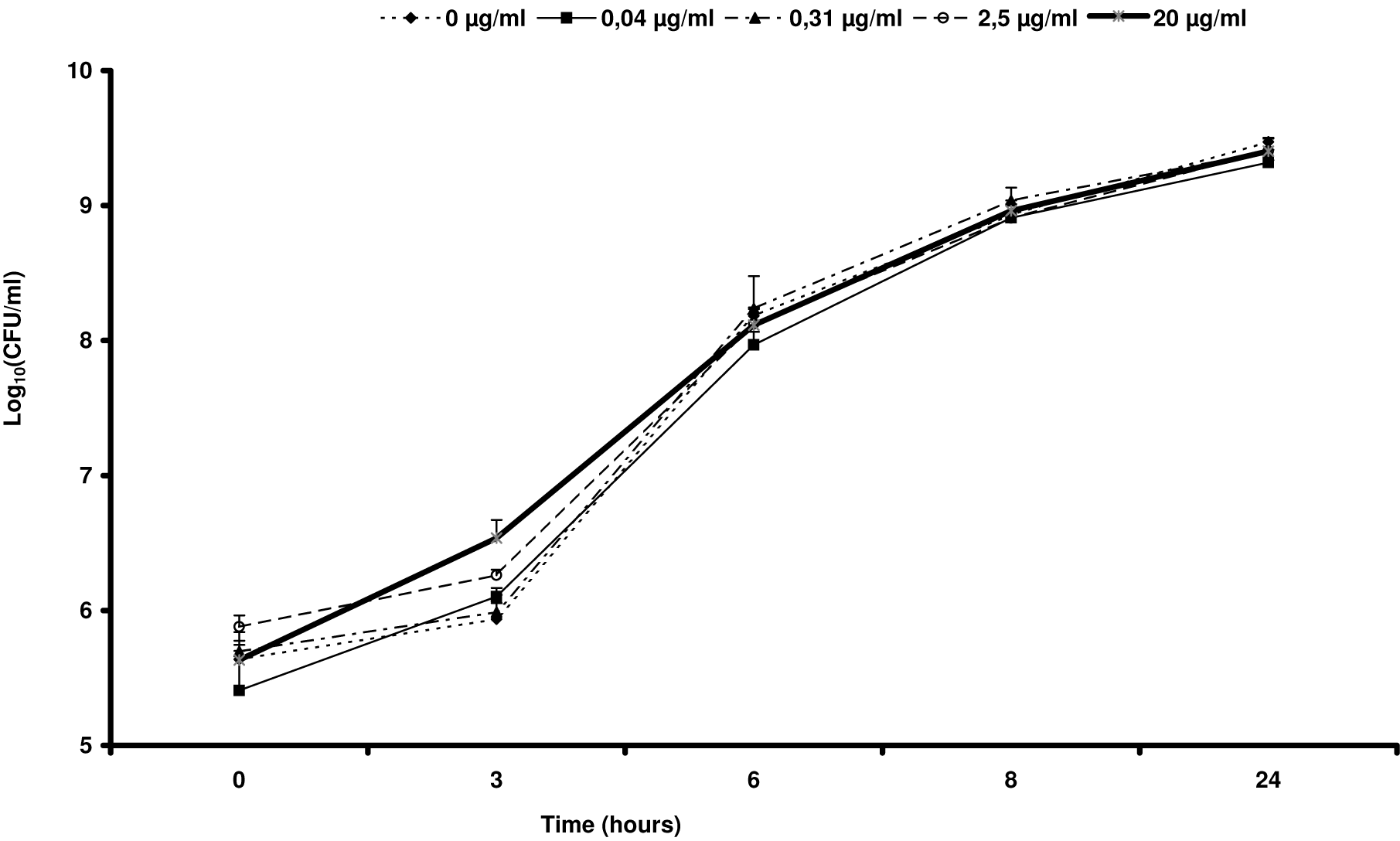

Supplement: Additional file 4 — Effect of T-2 toxin on the growth of Salmonella Typhimurium. The log10 values of the CFU/mL + standard deviation are given at different time points (t = 0, 2.5, 5, 7.5, 24 h). Salmonella Typhimurium growth was examined in LB medium, whether or not supplemented with T-2 toxin (0.04-20 μg/mL). Results are presented as a representative experiment conducted in triplicate. [file 1297-9716-43-22-S4.TIFF]
