# Supplementary material for: T-2 toxin induced Salmonella Typhimurium intoxication results in decreased Salmonella numbers in the cecum contents of pigs, despite marked effects on Salmonella-host cell interactions
Source: Vet Res. 2012 Mar 22;43(1):22. doi: 10.1186/1297-9716-43-22 (PMC3362764; doi:10.1186/1297-9716-43-22)
Supplement: Additional file 3 — TEER values of IPEC-J2 cells, 21 days after seeding them at density of 2 × 10^4^ cells, on collagen coated Transwell® polycarbonate membrane inserts. After 21 days, the cells were exposed to different concentrations of T-2 toxin ranging from 0 to 5 ng/mL, during 24 h. [file 1297-9716-43-22-S3.DOC]

| TEER values | At day 21 (Ohm/insert) | At day 22 (Ohm/insert) |
| --- | --- | --- |
| control 1 | 1650 | 1666 |
| control 2 | 1430 | 1532 |
| control 3 | 1510 | 1536 |
| 0,750 ng/mL T-2 toxin 1 | 1320 | 1465 |
| 0,750 ng/mL T-2 toxin 2 | 1489 | 1502 |
| 0,750 ng/mL T-2 toxin 3 | 1399 | 1466 |
| 1,0 ng/mL T-2 toxin 1 | 1678 | 1674 |
| 1,0 ng/mL T-2 toxin 2 | 1723 | 1836 |
| 1,0 ng/mL T-2 toxin 3 | 1985 | 2010 |
| 2,5 ng/mL T-2 toxin 1 | 1328 | 1401 |
| 2,5 ng/mL T-2 toxin 2 | 1504 | 1506 |
| 2,5 ng/mL T-2 toxin 3 | 1863 | 1935 |
| 5 ng/mL T-2 toxin 1 | 1652 | 1702 |
| 5 ng/mL T-2 toxin 2 | 1423 | 1536 |
| 5 ng/mL T-2 toxin 3 | 1489 | 1497 |
