# Supplementary material for: T-2 toxin induced Salmonella Typhimurium intoxication results in decreased Salmonella numbers in the cecum contents of pigs, despite marked effects on Salmonella-host cell interactions
Source: Vet Res. 2012 Mar 22;43(1):22. doi: 10.1186/1297-9716-43-22 (PMC3362764; doi:10.1186/1297-9716-43-22)
Supplement: Additional file 5 — Gene expression comparison between a logarithmic phase culture of Salmonella Typhimurium whether or not exposed to T-2 toxin. Microarray data of a logarithmic phase culture of Salmonella Typhimurium grown in presence or absence of 5 ng/mL T-2 toxin, showing genes differentially downregulated, by ≥ 1.5 fold with p ≤ 0.05. [file 1297-9716-43-22-S5.DOC]

| **STM** | **gene** | **description** | **fold change** |
| --- | --- | --- | --- |
| **Amino acid transport and metabolism** | | | |
| STM0316 | pepD | aminoacyl-histidine dipeptidase (peptidase D) | 0,59 |
| STM0365 | yahN | paral putative transport protein | 0,56 |
| STM0663 | gltK | ABC superfamily (membrane), glutamate/aspartate transporter | 0,65 |
| STM1057 | pepN | aminopeptidase N | 0,66 |
| STM1358 | aroD | 3-dehydroquinate dehydratase | 0,62 |
| STM1795 | STM1795 | putative homologue of glutamic dehyrogenase | 0,34 |
| STM1803 | dadA | D-amino acid dehydrogenase subunit | 0,41 |
| STM2354 | hisJ | ABC superfamily (bind_prot), histidine transport protein | 0,55 |
| STM2355 | argT | ABC superfamily (bind_prot), lysine/arginine/ornithine transport protein | 0,65 |
| STM2458 | eutB | ethanolamine ammonia-lyase, heavy chain | 0,46 |
| STM2536 | pepB | putative aminopeptidase | 0,57 |
| STM2555 | glyA | serine hydroxymethyltransferase | 0,60 |
| STM2970 | sdaC | putative HAAAP family, serine transport protein | 0,37 |
| STM3015 | ygeA | putative aspartate racemase | 0,66 |
| STM3054 | gcvH | glycine cleavage complex protein H, carrier of aminomethyl moiety via covalently bound lipoyl cofactor | 0,37 |
| STM3055 | gcvT | glycine cleavage complex protein T, aminomethyltransferase, tetrahydrofolate-dependent | 0,35 |
| STM3106 | ansB | periplasmic L-asparaginase II | 0,21 |
| STM3592 | yhiP | putative POT family, peptide transport protein | 0,49 |
| STM3904 | ilvD | dihydroxyacid dehydratase | 0,63 |
| STM3947 | dapF | diaminopimelate epimerase | 0,54 |
| STM3959 | rhtC | RhtB family, threonine efflux protein | 0,54 |
| STM4220 | lysC | aspartokinase III, lysine sensitive | 0,55 |
| STM4326 | aspA | aspartate ammonia-lyase (aspartase) | 0,47 |
| STM4469 | argI | ornithine carbamoyltransferase 1 | 0,60 |
| **Carbohydrate transport and metabolism** | | | |
| STM0007 | talB | transaldolase B | 0,47 |
| STM0684 | nagB | glucosamine-6-phosphate deaminase | 0,53 |
| STM0685 | nagE | Sugar Specific PTS family, n-acetylglucosamine-specific enzyme IIABC | 0,64 |
| STM1076 | mgsA | methylglyoxal synthase | 0,29 |
| STM1129 | STM1129 | N-acetylmannosamine-6-phosphate 2-epimerase | 0,32 |
| STM1467 | manA | mannose-6-phosphate isomerase | 0,52 |
| STM1830 | manX | Sugar Specific PTS family, mannose-specific enzyme IIAB | 0,43 |
| STM2083 | rfbK | LPS side chain defect: phosphomannomutase | 0,62 |
| STM2190 | mglB | ABC superfamily (peri_perm), galactose transport protein | 0,31 |
| STM2300 | STM2300 | putative cytoplasmic protein | 0,48 |
| STM2344 | STM2344 | PTS system, ascorbate-specific IIA component | 0,65 |
| STM2832 | srlA | PTS family, glucitol/sorbitol-specific enzyme IIC component,one of two IIC components | 0,48 |
| STM2974 | fucA | L-fuculose-1-phosphate aldolase | 0,61 |
| STM3259 | STM3259 | PTS family galactitol-specific enzyme IIB | 0,46 |
| STM3884 | rbsB | ABC superfamily (peri_perm), D-ribose transport protein | 0,51 |
| STM3963 | yigM | paral putative transport protein | 0,47 |
| STM4046 | rhaA | L-rhamnose isomerase | 0,57 |
| STM4054 | STM4054 | putative dicarboxylate-binding periplasmic protein | 0,57 |
| STM4078 | yneB | putative fructose-1,6-bisphosphate aldolase | 0,61 |
| STM4087 | glpF | MIP channel, glycerol diffusion | 0,21 |
| STM4230 | malK | bifunctional: ABC superfamily (atp_bind), maltose transportprotein; phenotypic repressor of mal operon | 0,60 |
| STM4468 | yjgK | putative cytoplasmic protein | 0,63 |
| **Carbohydrate transport and metabolism (Glycolysis)** | | | |
| STM0772 | gpmA | phosphoglyceromutase 1 | 0,54 |
| STM1326 | pfkB | 6-phosphofructokinase II | 0,56 |
| STM1378 | pykF | pyruvate kinase I (formerly F), fructose stimulated | 0,61 |
| STM1888 | pykA | pyruvate kinase II, glucose stimulated | 0,50 |
| STM2403 | glk | glucokinase | 0,51 |
| STM2952 | eno | enolase | 0,62 |
| STM3068 | fba | fructose-bisphosphate aldolase | 0,63 |
| STM4221 | pgi | glucosephosphate isomerase | 0,60 |
| **Cell motility and secretion** | | | |
| STM1172 | flgM | anti-FliA (anti-sigma) factor; also known as RflB protein | 0,54 |
| STM1183 | flgK | flagellar biosynthesis, hook-filament junction protein 1 | 0,53 |
| STM1184 | flgL | flagellar biosynthesis; hook-filament junction protein | 0,52 |
| STM1626 | trg | methyl-accepting chemotaxis protein III, ribose and galactose sensor receptor | 0,27 |
| STM1960 | fliD | flagellar biosynthesis; filament capping protein; enables filament assembly | 0,51 |
| STM2771 | fljB | Flagellar synthesis: phase 2 flagellin (filament structural protein) | 0,51 |
| STM3138 | STM3138 | putative methyl-accepting chemotaxis protein | 0,56 |
| **Cell envelope biogenesis and OM** | | | |
| STM0225 | hlpA | histone-like protein, located in outer membrane | 0,44 |
| STM0226 | lpxD | UDP-3-O-(3-hydroxymyristoyl)-glucosamine n-acyltransferase | 0,62 |
| STM1732 | ompW | outer membrane protein W; colicin S4 receptor; putative transporter | 0,44 |
| STM2080 | udg | UDP-glucose/GDP-mannose dehydrogenase | 0,42 |
| STM2089 | rfbJ | LPS side chain defect: CDP-abequose synthase | 0,61 |
| STM2090 | rfbH | LPS side chain defect: CDP-6deoxy-D-xylo-4-hexulose-3-dehydrase | 0,45 |
| STM2091 | rfbG | LPS side chain defect: CDP glucose 4,6-dehydratase | 0,52 |
| STM2092 | rfbF | LPS side chain defect: glucose-1-phosphate cytidylyltransferase | 0,45 |
| STM2298 | pmrF | putative glycosyl transferase | 0,65 |
| STM2299 | yfbG | paral putative transformylase | 0,43 |
| STM2301 | pqaB | putative melittin resistance protein, PqaB, affects polymycin B resistance and lipopolysaccharide synthesis | 0,63 |
| **Coenzyme metabolism** | | | |
| STM2018 | cobU | bifunctional: cobinamide kinase; cobinamide phosphate guanylyltransferase | 0,57 |
| STM3090 | metK | methionine adenosyltransferase 1 (AdoMet synthetase) | 0,46 |
| STM3849 | yieE | putative cytoplasmic protein | 0,58 |
| **Energy production and conversion** | | | |
| STM0154 | lpdA | lipoamide dehydrogenase (NADH); component of 2-oxodehydrogenase and pyruvate complexes; L protein of glycine cleavage complex second part | 0,44 |
| STM0158 | acnB | aconitate hydratase 2 | 0,43 |
| STM0441 | cyoC | cytochrome o ubiquinol oxidase subunit III | 0,41 |
| STM0442 | cyoB | cytochrome o ubiquinol oxidase subunit I | 0,43 |
| STM0443 | cyoA | cytochrome o ubiquinol oxidase subunit II | 0,39 |
| STM0730 | gltA | citrate synthase | 0,28 |
| STM0732 | sdhC | succinate dehydrogenase, cytochrome b556 | 0,41 |
| STM0733 | sdhD | succinate dehydrogenase, hydrophobic subunit | 0,29 |
| STM0734 | sdhA | succinate dehydrogenase, flavoprotein subunit | 0,37 |
| STM0735 | sdhB | succinate dehydrogenase, Fe-S protein | 0,55 |
| STM0741 | cydB | cytochrome d terminal oxidase polypeptide subunit II | 0,55 |
| STM0973 | pflB | pyruvate formate lyase I, induced anaerobically | 0,43 |
| STM1124 | putA | bifunctional in plasma membrane proline dehydrogenase and pyrroline-5-carboxylate dehydrogenase OR in cytoplasm a transcriptional repressor | 0,58 |
| STM1238 | icdA | isocitrate dehydrogenase in e14 prophage, specific for NADP+ | 0,49 |
| STM1296 | ydjA | putative oxidoreductase | 0,53 |
| STM1468 | fumA | fumarase A (fumarate hydratase class I), aerobic isozyme | 0,37 |
| STM1570 | fdnG | putative molybdopterin oxidoreductases | 0,52 |
| STM2039 | pudB | Propanediol utilization: polyhedral bodies | 0,50 |
| STM2051 | pduP | Propanediol utilization: CoA-dependent propionaldehyde dehydrogenase | 0,52 |
| STM2064 | phsB | Hydrogen sulfide production: iron- sulfur subunit; electron transfer | 0,59 |
| STM2316 | nuoN | NADH dehydrogenase I chain N | 0,56 |
| STM2317 | nuoM | NADH dehydrogenase I chain M | 0,58 |
| STM2318 | nuoL | NADH dehydrogenase I chain L | 0,61 |
| STM2320 | nuoJ | NADH dehydrogenase I chain J | 0,62 |
| STM2322 | nuoH | NADH dehydrogenase I chain H | 0,62 |
| STM2326 | nuoC | NADH dehydrogenase I chain C,D | 0,59 |
| STM2327 | nuoB | NADH dehydrogenase I chain B | 0,56 |
| STM2337 | ackA | acetate kinase A (propionate kinase 2) | 0,52 |
| STM2338 | pta | phosphotransacetylase | 0,57 |
| STM2472 | maeB | paral putative transferase | 0,58 |
| STM2538 | fdx | [2FE-2S] ferredoxin, electron carrer protein, believed to be involved in assembly of Fe-S clusters | 0,49 |
| STM2845 | hycI | protease involved in processing C-terminal end of HycE | 0,47 |
| STM2847 | hycG | hydrogenase activity | 0,46 |
| STM3081 | STM3081 | putative malate/L-lactate dehydrogenase | 0,61 |
| STM3500 | pckA | phosphoenolpyruvate carboxykinase | 0,36 |
| STM3614 | dctA | DAACS family, C4-dicarboxylic acids transport protein | 0,39 |
| STM3865 | atpD | membrane-bound ATP synthase, F1 sector, beta-subunit | 0,53 |
| STM3867 | atpA | membrane-bound ATP synthase, F1 sector, alpha-subunit | 0,65 |
| STM3870 | atpE | membrane-bound ATP synthase, F0 sector, subunit c | 0,60 |
| STM4036 | fdoH | formate dehydrogenase-O, Fe-S subunit | 0,50 |
| STM4037 | fdoG | formate dehydrogenase | 0,42 |
| STM4300 | fumB | fumarase B (fumarate hydratase class I), anaerobic isozyme | 0,62 |
| **Inorganic ion transport and metabolism** | | | |
| STM0245 | yaeC | putative outer membrane lipoprotein | 0,65 |
| STM1806 | nhaB | NhaB family of transport protein, Na+/H+ antiporter, regulator of intracellular pH | 0,63 |
| STM3505 | feoA | ferrous iron transport protein A | 0,42 |
| STM3506 | feoB | FeoB family, ferrous iron transport protein B | 0,57 |
| STM4055 | sodA | superoxide dismutase, manganese | 0,58 |
| **Nucleotide transport and metabolism** | | | |
| STM0515 | allA | ureidoglycolate hydrolase | 0,56 |
| STM0661 | ybeK | putative purine nucleoside hydrolase | 0,64 |
| STM2278 | nrdB | ribonucleoside-diphosphate reductase 1, beta subunit | 0,54 |
| STM2437 | yfeJ | putative GMP synthase - glutamine amidotransferase domain | 0,54 |
| STM3167 | STM3167 | putative diadenosine tetraphosphate (Ap4A) hydrolase | 0,56 |
| STM4366 | purA | adenylosuccinate synthetase | 0,62 |
| STM4403 | cpdB | 2':3'-cyclic-nucleotide 2'-phosphodiesterase | 0,50 |
| **Signal transduction** | | | |
| STM0600 | cstA | carbon starvation protein | 0,43 |
| STM0614 | ybdQ | putative Universal stress protein UspA and related nucleotide-binding protein | 0,41 |
| STM1652 | ynaF | putative universal stress protein | 0,50 |
| STM1661 | ydaA | putative universal stress protein | 0,46 |
| STM2314 | STM2314 | putative chemotaxis signal transduction protein | 0,28 |
| STM3217 | aer | aerotaxis sensor receptor, senses cellular redox state or proton motive force | 0,45 |
| STM3466 | crp | catabolite activator protein (CAP), cyclic AMP receptor protein (CRP family) | 0,63 |
| STM3591 | uspA | universal stress protein A | 0,54 |
| STM4292 | basR | response regulator in two-component regulatory system with BasS (OmpR family) | 0,66 |
| **SPI1** | | | |
| STM2870 | STM2870 | oxygen-regulated invasion protein ORGA. (SW:ORGA_SALTY); putative inner membrane protein [Salmonella typhimurium LT2]. | 0,62 |
| STM2871 | prgK | lipoprotein; may link inner and outer membranes; PRGK protein precursor. (SW:PRGK_SALTY); cell invasion protein [Salmonella typhimurium LT2]. | 0,59 |
| STM2872 | prgJ | Putative RBS for prgJ; RegulonDB:STMS1H002936 | 0,51 |
| STM2878 | sptP | protein tyrosine phosphatase SptP (gi|1519054); protein tyrosine phosphate [Salmonella typhimurium LT2]. | 0,49 |
| STM2879 | sicP | SicP (gi|3283218); chaparone related to virulence [Salmonella typhimurium LT2]. | 0,63 |
| STM2880 | STM2880 | hypothetical protein; putative cytoplasmic protein [Salmonella typhimurium LT2]. | 0,60 |
| STM2881 | iacP | acyl carrier protein (gi|1172129); putative acyl carrier protein [Salmonella typhimurium LT2]. | 0,56 |
| STM2882 | sipA | SipA (gi|1172128); cell invasion protein [Salmonella typhimurium LT2]. | 0,48 |
| STM2884 | sipC | sspC protein (gi|7443298); cell invasion protein [Salmonella typhimurium LT2]. | 0,61 |
| STM2886 | sicA | secretory proteins; surface presentation of antigens protein SPAT. (SW:SPAT_SALTY); surface presentation of antigens [Salmonella typhimurium LT2]. | 0,55 |
| STM2890 | spaP | Putative RBS for spaP; RegulonDB:STMS1H002952 | 0,53 |
| STM2892 | invJ | secretory proteins; surface presentation of antigens protein SPAN. (SW:SPAN_SALTY); surface presentation of antigens [Salmonella typhimurium LT2]. | 0,51 |
| STM2893 | invI | secretory proteins; surface presentation of antigens protein SPAM. (SW:SPAM_SALTY); surface presentation of antigens [Salmonella typhimurium LT2]. | 0,57 |
| STM2894 | invC | secretory proteins; probable ATP synthase SPAL. (SW:SPAL_SALTY); surface presentation of antigens [Salmonella typhimurium LT2]. | 0,53 |
| STM2895 | invB | secretory proteins; surface presentation of antigens protein SPAK. (SW:SPAK_SALTY); surface presentation of antigens [Salmonella typhimurium LT2]. | 0,54 |
| STM2900 | invH | invasion protein INVH precursor. (SW:INVH_SALTY); invasion protein [Salmonella typhimurium LT2]. | 0,66 |
| **SPI2** | | | |
| STM1380 | orf32 | proline iminopeptidase like protein (gi|1526980); putative hydrolase or acyltransferase [Salmonella typhimurium LT2]. | 0,63 |
| STM1396 | ssaE | putative secretory apparatus component (gi|3377860); secretion system effector [Salmonella typhimurium LT2]. | 0,51 |
| STM2533 | sseA | similar to E. coli putative thiosulfate sulfurtransferase (AAC75574.1); Blastp hit to AAC75574.1 (334 aa), 80% identity in aa 54 - 332; putative sulfurtransferase [Salmonella typhimurium LT2]. | 0,52 |
| **SPI3** | | | |
| STM3762 | cigR | Pathogenicity island encoded protein: SPI3; CigR (gi|4324614); putative inner membrane protein [Salmonella typhimurium LT2]. | 0,56 |
| **SPI4** | | | |
| STM4260 | STM4260 | predicted cation efflux pump; HLYD secretion protein (gi|7467244); membrane permease [Salmonella typhimurium LT2]. | 0,54 |
| **SPI5** | | | |
| STM1090 | pipC | invasion gene E protein (gi|2582386); Pathogenicity island encoded protein: SPI5 [Salmonella typhimurium LT2]. | 0,60 |
| STM1092 | orfX | Pathogenicity island encoded protein: SPI5 [Salmonella typhimurium LT2]. | 0,31 |
| **Islands (not covered in other groups)** | | | |
| STM0328 | STM0328 | Paralog of E. coli putative transport protein (AAC74668.1); Blastp hit to AAC74668.1 (417 aa), 34% identity in aa 33 - 407 | 0,64 |
| STM0332 | STM0332 | similar to E. coli orf, hypothetical protein (AAC73780.1); Blastp hit to AAC73780.1 (254 aa), 33% identity in aa 15 - 129; putative hydrolase or acyltransferase [Salmonella typhimurium LT2]. | 0,64 |
| STM0905 | STM0905 | similar to E. coli orf, hypothetical protein (AAC74261.1); Blastp hit to AAC74261.1 (122 aa), 48% identity in aa 3 - 118; Fels-1 prophage hypothetical protein [phage Fels-1]. | 0,65 |
| STM0906 | STM0906 | Fels-1 prophage protein [phage Fels-1]. | 0,42 |
| STM0907 | STM0907 | putative Fels-1 prophage chitinase [phage Fels-1]. | 0,60 |
| STM0912 | STM0912 | similar to E. coli ATP-dependent proteolytic subunit of clpA-clpP serine protease; ClpP family members; heat shock protein F21.5 (AAC73540.1); Blastp hit to AAC73540.1 (207 aa), 29% identity in aa 73 - 203; Fels-1 prophage protease subunits of ATP-dependent proteases [phage Fels-1]. | 0,53 |
| STM1018 | STM1018 | S. typhimurium hypothetical protein 11 (gi|7467261) | 0,65 |
| STM1032 | STM1032 | Putative RBS for STM1032; RegulonDB:STMS1H001312 | 0,51 |
| STM1034 | STM1034 | Gifsy-2 prophage putative RecA/RadA recombinase [phage Gifsy-2]. | 0,55 |
| STM1039 | STM1039 | Gifsy-2 prophage probable minor tail protein [phage Gifsy-2]. | 0,38 |
| STM1043 | STM1043 | attachment and invasion protein homolog (gi|2232362); Gifsy-2 prophage attachment and invasion protein homolog [phage Gifsy-2]. | 0,66 |
| STM1116 | scsD | S. typhimurium suppressor for copper-sensitivity D (gi|2327006) | 0,64 |
| STM1133 | STM1133 | similar to E. coli putative dehydrogenase (AAC77236.1); Blastp hit to AAC77236.1 (377 aa), 62% identity in aa 6 - 371; putative dehydrogenases and related proteins [Salmonella typhimurium LT2]. | 0,54 |
| STM1249 | STM1249 | putative periplasmic protein [Salmonella typhimurium LT2]. | 0,55 |
| STM1329 | STM1329 | hypothetical protein; putative inner membrane protein [Salmonella typhimurium LT2]. | 0,62 |
| STM1554 | STM1554 | putative coiled-coil protein [Salmonella typhimurium LT2]. | 0,63 |
| STM1854 | STM1854 | hypothetical protein; putative inner membrane protein [Salmonella typhimurium LT2]. | 0,55 |
| STM1855 | sopE2 | SopE2 (gi|9651642); TypeIII-secreted protein effector: invasion-associated protein [Salmonella typhimurium LT2]. | 0,54 |
| STM1919 | cheM | aspartate sensor-receptor; methyl-accepting chemotaxis protein II (MCP-II). (SW:MCP2_SALTY); methyl accepting chemotaxis protein II [Salmonella typhimurium LT2]. | 0,51 |
| STM2038 | pduA | polyhedral bodies; propanediol utilization protein PDUA. (SW:PDUA_SALTY); propanediol utilization protein [Salmonella typhimurium LT2]. | 0,48 |
| STM2045 | pduJ | polyhedral bodies; similar to E. coli detox protein (AAC75510.1); Blastp hit to AAC75510.1 (111 aa), 64% identity in aa 17 - 101; propanediol utilization protein [Salmonella typhimurium LT2]. | 0,56 |
| STM2046 | pduK | polyhedral bodies; similar to E. coli detox protein (AAC75510.1); Blastp hit to AAC75510.1 (111 aa), 35% identity in aa 9 - 92; propanediol utilization protein [Salmonella typhimurium LT2]. | 0,59 |
| STM2047 | pduL | propanediol utilization protein [Salmonella typhimurium LT2]. | 0,65 |
| STM2178 | STM2178 | Putative RBS for STM2178; RegulonDB:STMS1H002321 | 0,64 |
| STM2238 | STM2238 | putative phage protein [Salmonella typhimurium LT2]. | 0,54 |
| STM2603 | STM2603 | similar to head protein gp7 of phage 21; Gifsy-1 prophage protein [Salmonella typhimurium LT2]. | 0,65 |
| STM2605 | STM2605 | similar to head-tail preconnector gp5 of phage 21; similar to E. coli putative protease (AAC74354.1); Blastp hit to AAC74354.1 (349 aa), 27% identity in aa 124 - 300; Gifsy-1 prophage protein [Salmonella typhimurium LT2]. | 0,66 |
| STM2621 | STM2621 | hypothetical protein 13 (gi|7467262); Gifsy-1 prophage protein [Salmonella typhimurium LT2]. | 0,61 |
| STM2694 | STM2694 | Putative RBS for STM2694; RegulonDB:STMS1H002780 | 0,61 |
| STM2700 | STM2700 | Putative RBS for STM2700; RegulonDB:STMS1H002786 | 0,45 |
| STM2718 | STM2718 | Putative RBS for STM2718; RegulonDB:STMS1H002803 | 0,60 |
| STM2723 | STM2723 | similar to gpQ; portal vector protein in phage P2; Fels-2 prophage protein [phage Fels-2]. | 0,42 |
| STM2730 | STM2730 | similar to retron in E coli; similar to E. coli DNA adenine methylase (AAC76412.1); Blastp hit to AAC76412.1 (278 aa), 46% identity in aa 8 - 266; Fels-2 prophage protein [Salmonella typhimurium LT2]. | 0,61 |
| STM2763 | STM2763 | similar to E. coli prophage CP4-57 integrase (AAC75670.1); Blastp hit to AAC75670.1 (413 aa), 47% identity in aa 1 - 185; putative integrase [Salmonella typhimurium LT2]. | 0,59 |
| STM2787 | STM2787 | tricarboxylic transport protein [Salmonella typhimurium LT2]. | 0,43 |
| STM3119 | STM3119 | similar to E. coli putative aldehyde dehydrogenase (AAC74469.1); Blastp hit to AAC74469.1 (681 aa), 29% identity in aa 530 - 661; putative monoamine oxidase [Salmonella typhimurium LT2]. | 0,65 |
| STM3197 | glgS | similar to E. coli glycogen biosynthesis, rpoS dependent (AAC76085.1); Blastp hit to AAC76085.1 (66 aa), 78% identity in aa 2 - 66; rpoS dependent glycogen biosynthesis protein [Salmonella typhimurium LT2]. | 0,41 |
| STM4013 | STM4013 | putative membrane-associated metal-dependent hydrolase [Salmonella typhimurium LT2]. | 0,66 |
| STM4200 | STM4200 | similar to E. coli putative membrane protein (AAC74454.1); Blastp hit to AAC74454.1 (1122 aa), 31% identity in aa 312 - 498, 33% identity in aa 426 - 547, 22% identity in aa 233 - 444, 30% identity in aa 132 - 266, 23-1073758628dentity in aa 170 - 398, 21% identity in aa 178 - 417, 24-1073758654dentity in aa 111 - 354, 23% identity in aa 950 - 1104, 20% identity in aa 1015 - 1106; putative phage tail fiber protein H [Salmonella typhimurium LT2]. | 0,65 |
| STM4214 | STM4214 | Putative RBS for STM4214; RegulonDB:STMS1H004121 | 0,54 |
| **Surface structure** | | | |
| STM0319 | crl | transcriptional regulator of cryptic csgA gene for curli surface fibers | 0,66 |
| STM2152 | stcA | paral putative fimbrial-like protein | 0,60 |
| **Translation, ribosomal structure and biogenesis** | | | |
| STM1167 | rimJ | acetylation of N-terminal alanine of 30S ribosomal subunit protein S5 | 0,51 |
| STM1334 | infC | protein chain initiation factor IF-3 | 0,58 |
| STM1502 | speG | spermidine N1-acetyltransferase | 0,56 |
| STM2665 | yfiA | ribosome associated factor, stabilizes ribosomes against dissociation | 0,18 |
| STM2674 | trmD | tRNA (guanine-7-)-methyltransferase | 0,51 |
| STM2675 | rimM | 16S rRNA processing protein | 0,59 |
| STM3282 | pnp | polynucleotide phosphorylase, member of mRNA degradosome | 0,47 |
| STM3283 | rpsO | 30S ribosomal subunit protein S15 | 0,61 |
| STM3284 | truB | tRNA pseudouridine 5S synthase | 0,41 |
| STM3285 | rbfA | ribosome-binding factor, role in processing of 10S rRNA | 0,34 |
| STM3344 | rpsI | 30S ribosomal subunit protein S9 | 0,54 |
| STM3414 | rplQ | 50S ribosomal subunit protein L17 | 0,62 |
| STM3417 | rpsK | 30S ribosomal subunit protein S11 | 0,54 |
| STM3419 | rpmJ | 50S ribosomal subunit protein X | 0,51 |
| STM3421 | rplO | 50S ribosomal subunit protein L15 | 0,29 |
| STM3423 | rpsE | 30S ribosomal subunit protein S5 | 0,40 |
| STM3424 | rplR | 50S ribosomal subunit protein L18 | 0,32 |
| STM3427 | rpsN | 30S ribosomal subunit protein S14 | 0,46 |
| STM3431 | rpsQ | 30S ribosomal subunit protein S17 | 0,31 |
| STM3432 | rpmC | 50S ribosomal subunit protein L29 | 0,40 |
| STM3433 | rplP | 50S ribosomal subunit protein L16 | 0,28 |
| STM3434 | rpsC | 30S ribosomal subunit protein S3 | 0,20 |
| STM3435 | rplV | 50S ribosomal subunit protein L22 | 0,27 |
| STM3436 | rpsS | 30S ribosomal subunit protein S19 | 0,27 |
| STM3437 | rplB | 50S ribosomal subunit protein L2 | 0,32 |
| STM3438 | rplW | 50S ribosomal subunit protein L23 | 0,26 |
| STM3439 | rplD | 50S ribosomal subunit protein L4, regulates expression of S10 operon | 0,34 |
| STM3445 | tufA | protein chain elongation factor EF-Tu (duplicate of tufB) | 0,46 |
| STM3446 | fusA | protein chain elongation factor EF-G, GTP-binding | 0,46 |
| STM3448 | rpsL | 30S ribosomal subunit protein S12 | 0,62 |
| STM4028 | yihZ | D-Tyr-tRNA(Tyr) deacylase | 0,64 |
| STM4146 | tufB | protein chain elongation factor EF-Tu (duplicate of tufA) | 0,39 |
| STM4391 | rpsF | 30S ribosomal subunit protein S6 | 0,43 |
| STM4393 | rpsR | 30S ribosomal subunit protein S18 | 0,55 |
| STM4394 | rplI | 50S ribosomal subunit protein L9 | 0,42 |
| STM4458 | yjgF | putative translation initiation inhibitor | 0,44 |
| **Transcription** | | | |
| STM0031 | STM0031 | putative transcription regulator | 0,47 |
| STM0430 | phnR | 2-aminoethylphosphonate transport, repressor | 0,64 |
| STM0516 | allR | putative regulatory protein | 0,61 |
| STM0629 | cspE | RNA chaperone, negative regulator of cspA transcription | 0,58 |
| STM0763 | STM0763 | transcriptional regulator, lysR family | 0,65 |
| STM0959 | lrp | regulator for lrp regulon and high-affinity branched-chain amino acid transport system; mediator of of leucine response (AsnC family) | 0,65 |
| STM1213 | ycfQ | putative transcriptional repressor (TetR/AcrR family) | 0,66 |
| STM1837 | cspC | cold shock protein, multicopy suppresses mukB mutants, putative regulator | 0,46 |
| STM3011 | galR | transcriptional repressor of galETK operon (GalR/LacI family) | 0,61 |
| STM3262 | STM3262 | transcriptional regulator of sugar metabolism | 0,59 |
| STM3389 | envR | transcriptional repressor for envCD (acrEF) (TetR/AcrR family) | 0,16 |
| STM3415 | rpoA | RNA polymerase, alpha subunit | 0,42 |
| STM4049 | rhaR | positive regulator for rhaRS operon (AraC/XylS familiy) | 0,50 |
| STM4297 | melR | regulator of melibiose operon (AraC/XylS family) | 0,57 |
| STM4423 | STM4423 | putative AraC-type DNA-binding domain-containing protein | 0,66 |
| **Unknown function** | | | |
| STM0160 | yacL | putative cytoplasmic protein | 0,51 |
| STM0258 | yafD | putative cytoplasmic protein | 0,58 |
| STM0587 | ybdZ | putative cytoplasmic protein | 0,50 |
| STM1168 | yceH | putative cytoplasmic protein | 0,59 |
| STM2390 | yfcZ | putative cytoplasmic protein | 0,62 |
| STM3176 | ygiW | putative outer membrane protein | 0,50 |
| STM3332 | yhcG | putative cytoplasmic protein | 0,48 |
| STM4088 | yiiU | putative cytoplasmic protein | 0,26 |
