# Supplementary material for: T-2 toxin induced Salmonella Typhimurium intoxication results in decreased Salmonella numbers in the cecum contents of pigs, despite marked effects on Salmonella-host cell interactions
Source: Vet Res. 2012 Mar 22;43(1):22. doi: 10.1186/1297-9716-43-22 (PMC3362764; doi:10.1186/1297-9716-43-22)
Supplement: Additional file 6 — Gene expression comparison between a logarithmic phase culture of Salmonella Typhimurium whether or not exposed to T-2 toxin. Microarray data of a logarithmic phase culture of Salmonella Typhimurium grown in presence or absence of 5 ng/mL T-2 toxin, showing genes differentially upregulated, by ≥ 1.5 fold with p ≤ 0.05. [file 1297-9716-43-22-S6.DOC]

| **STM** | **gene** | **description** | **fold change** |
| --- | --- | --- | --- |
| **Amino acid transport and metabolism** | | | |
| STM0006 | yaaJ | putative AGCS family, alanine/glycine transport protein | 1,56 |
| STM0399 | brnQ | LIVCS family, branched chain amino acid transporter system II (LIV-II) | 2,18 |
| STM0426 | phnV | 2-aminoethylphosphonate transporter, membrane component | 2,73 |
| STM0532 | arcC | putative carbamate kinase | 1,74 |
| STM0710 | ybgH | putative POT family transport protein | 1,69 |
| STM0849 | yliB | putative ABC transporter periplasmic binding protein | 1,57 |
| STM0850 | yliC | putative ABC transporter periplasmic binding protein | 2,01 |
| STM0851 | yliD | putative ABC transporter inner membrane component | 1,84 |
| STM0978 | aroA | 3-enolpyruvylshikimate-5-phosphate synthetase | 1,53 |
| STM1278 | yeaN | putative MFS family transport protein (amino acid/amine transport) | 1,63 |
| STM1347 | aroH | 3-deoxy-D-arabinoheptulosonate-7-phosphate synthase (DAHP synthetase), tryptophan repressible | 1,71 |
| STM1373 | sufS | selenocysteine lyase | 1,52 |
| STM1452 | ydgR | putative POT family, peptide transport protein | 2,98 |
| STM1477 | ydgI | putative amino acid transporter | 1,55 |
| STM1937 | tyrP | HAAAP family, tyrosine-specific transport protein | 1,77 |
| STM2162 | yehW | putative ABC-type proline/glycine betaine transport systems, permease component | 1,96 |
| STM2163 | yehX | putative ABC-type proline/glycine betaine transport system, ATPase component | 2,72 |
| STM2164 | yehY | putative ABC-type proline/glycine betaine transport systems, permease component | 2,14 |
| STM2186 | STM2186 | putative NADPH-dependent glutamate synthase beta chain or related oxidoreductase | 1,61 |
| STM2216 | yejA | putative ABC transporter periplasmic binding protein | 1,53 |
| STM2402 | yfdZ | putative aminotransferase | 1,58 |
| STM2809 | proV | ABC superfamily (atp_bind), glycine/betaine/proline transport protein | 2,00 |
| STM2984 | csdA | putative selenocysteine lyase | 3,66 |
| STM3013 | lysA | diaminopimelate decarboxylase | 1,64 |
| STM3022 | STM3022 | putative transport protein | 2,11 |
| STM3129 | STM3129 | putative NAD-dependent aldehyde dehydrogenase | 2,05 |
| STM3401 | aroE | dehydroshikimate reductase | 2,26 |
| STM3468 | argD | acetylornithine transaminase (NAcOATase and DapATase) | 1,68 |
| STM3469 | pabA | p-aminobenzoate synthetase, component II | 1,59 |
| STM3567 | livJ | ABC superfamily (bind_prot), branched-chain amino acid transporter, high-affinity | 1,93 |
| STM3625 | yhjV | putative HAAAP family transport protein | 1,83 |
| STM3877 | asnA | asparagine synthetase A | 1,88 |
| STM4345 | yjeM | putative APC family, amino-acid transport protein | 1,84 |
| STM4398 | cycA | APC family, D-alanine/D-serine/glycine transport protein | 2,23 |
| **Carbohydrate transport and metabolism** | | | |
| STM0401 | malZ | maltodextrin glucosidase | 1,56 |
| STM0491 | gsk | inosine-guanosine kinase | 2,11 |
| STM0493 | fsr | putative MFS family of transport protein | 1,51 |
| STM0681 | nagD | putative phosphatase in N-acetylglucosamine metabolism | 1,84 |
| STM0860 | STM0860 | putative inner membrane protein | 2,97 |
| STM0861 | yliI | putative dehydrogenase | 2,23 |
| STM0968 | ycaD | putative MFS family transport protein | 1,89 |
| STM1252 | STM1252 | putative sialidase | 2,00 |
| STM1486 | ynfM | putative MFS familty transport protein | 2,34 |
| STM1516 | ydeE | putative MFS family transport protein | 2,18 |
| STM1560 | STM1560 | putative alpha amylase | 2,10 |
| STM1708 | yciM | putative N-acetylglucosaminyl transferase | 1,61 |
| STM1843 | STM1843 | putative transport protein | 1,51 |
| STM1928 | otsA | trehalose-6-phosphate synthase | 2,41 |
| STM2280 | STM2280 | putative permease | 2,04 |
| STM2654 | kgtP | MFS family, alpha-ketoglutarate permease | 2,29 |
| STM3324 | ptsO | NPr, phosphocarrier protein HPr-like NPr, nitrogen related, exchanges phosphate with Enzyme I | 2,58 |
| STM3537 | glgX | glycosyl hydrolase | 2,00 |
| STM3538 | glgB | 1,4-alpha-glucan branching enzyme | 1,70 |
| STM3554 | ugpC | ABC superfamily (atp_bind), sn-glycerol 3-phosphate transport protein | 1,86 |
| STM3556 | ugpA | ABC superfamily (membrane), sn-glycerol 3-phosphate transport protein | 1,98 |
| STM3603 | treF | cytoplasmic trehalase | 1,61 |
| STM3609 | yhjE | putative MFS family transport protein | 2,48 |
| STM3664 | malS | alpha-amylase | 1,55 |
| STM3698 | STM3698 | putative permease | 2,95 |
| STM3765 | yicL | putative permease, integral membrane protein | 1,52 |
| STM3776 | yicM | putative MFS family tranport protein (1st mdule) | 2,54 |
| STM3827 | dgoT | MFS family, D-galactonate transport protein | 2,31 |
| STM4019 | yihQ | putative alpha-xylosidase | 1,58 |
| STM4116 | frwD | PTS system fructose-like IIB component 2 | 1,68 |
| STM4290 | proP | MFS family, low-affinity proline transporter (proline permease II) | 3,82 |
| STM4412 | STM4412 | putative pemease | 2,13 |
| STM4418 | STM4418 | sugar (and other) transporter | 2,60 |
| STM4515 | yjiJ | putative sugar transporter | 1,51 |
| STM4517 | yjiO | putative MFS family transport protein | 2,23 |
| **Cell motility and secretion** | | | |
| STM1980 | fliQ | flagellar biosynthesis | 1,76 |
| **Cell envelope biogenesis and OM** | | | |
| STM0123 | murE | UDP-N-acetylmuramoylalanyl-D-glutamate 2,6-diaminopimelate ligase | 1,85 |
| STM0124 | murF | D-alanine:D-alanine-adding enzyme | 1,56 |
| STM0125 | mraY | phospho-N-acetylmuramoyl-pentapeptide transferase | 1,77 |
| STM0126 | murD | UDP-N-acetylmuramoylalanine-D-glutamate ligase | 1,78 |
| STM0128 | murG | UDP-N-acetylglucosamine:N-acetylmuramyl-(pentapeptide) pyrophosphoryl-undecaprenol N-acetylglucosamine transferase | 1,80 |
| STM0260 | dniR | transcriptional regulator for nitrite reductase (cytochrome c552) | 2,86 |
| STM0535 | lpxH | UDP-2,3-diacylglucosamine hydrolase | 1,83 |
| STM0559 | rfbI | bactoprenol-linked glucose translocase | 1,84 |
| STM0637 | dacA | D-alanyl-D-alanine carboxypeptidase, penicillin-binding protein 5 | 1,79 |
| STM0666 | lnt | apolipoprotein N-acyltransferase, copper homeostasis protein, inner membrane | 2,86 |
| STM0827 | ybiO | paral putative transport protein | 1,55 |
| STM0985 | lpxK | tetraacyldisaccharide 4' kinase (lipid A 4'kinase) | 1,59 |
| STM1217 | ycfU | ABC transporter, integral membrane protein | 1,69 |
| STM1343 | nlpC | lipoprotein | 1,73 |
| STM1845 | prc | carboxy-terminal protease for penicillin-binding protein 3 | 1,57 |
| STM1890 | yebA | putative Peptidase | 1,60 |
| STM1910 | STM1910 | putative penicillin-binding protein | 1,83 |
| STM2079 | wzzB | regulator of length of O-antigen component of lipopolysaccharide chains | 2,13 |
| STM2098 | galF | putative glucose-1-phosphate uridylyltransferase (UDP-glucose pyrophosphorylase), non-catalytic subunit | 1,69 |
| STM2120 | asmA | suppressor of ompF assembly mutants | 1,80 |
| STM2439 | yfeL | putative membrane carboxypeptidase (penicillin-binding protein) | 2,72 |
| STM2546 | suhB | inositol monophosphatase | 5,62 |
| STM2450 | amiA | N-acetylmuramoyl-l-alanine amidase I | 2,03 |
| STM2567 | yfhD | putative periplasmic amino acid binding protein | 1,72 |
| STM2831 | mltB | membrane-bound lytic murein transglycosylase B | 1,52 |
| STM2991 | amiC | N-acetylmuramoyl-L-alanine amidase | 1,57 |
| STM3002 | lgt | phosphatidylglycerol-prolipoprotein diacylglyceryl transferase | 1,75 |
| STM3268 | yraR | putative nucleoside-diphosphate-sugar epimerase | 1,94 |
| STM3372 | mreD | rod shape-determining protein | 1,85 |
| STM3712 | rfaC | heptosyl transferase I | 1,85 |
| STM3724 | kdtA | 3-deoxy-D-manno-octulosonic-acid transferase (KDO transferase) | 2,86 |
| STM3833 | STM3833 | putative mandelate racemase / muconate lactonizing enzyme family | 1,68 |
| STM3873 | gidB | associate with glucose-inhibited division | 1,83 |
| STM4102 | STM4102 | putative inner membrane protein | 1,86 |
| STM4339 | blc | outer membrane lipoprotein (lipocalin) | 2,20 |
| STM4416 | mpl | UDP-N-acetylmuramate:L-alanyl-gamma-D-glutamyl-meso-diaminopimelate ligase | 1,56 |
| **Coenzyme metabolism** | | | |
| STM0087 | folA | dihydrofolate reductase type I; trimethoprim resistance | 2,84 |
| STM0106 | thiQ | thiamine transporter ATP-binding subunit | 2,64 |
| STM0183 | folK | 7,8-dihydro-6-hydroxymethylpterin-pyrophosphokinase, PPPK | 1,99 |
| STM0422 | dxs | 1-deoxyxylulose-5-phosphate synthase; flavoprotein | 1,61 |
| STM0757 | pnuC | NMN family, nucleoside/purine/pyrimidine transporter | 1,69 |
| STM0802 | moaA | molybdopterin biosynthesis, protein A | 1,77 |
| STM0804 | moaC | molybdopterin biosynthesis, protein C | 1,59 |
| STM0806 | moaE | molybdopterin converting factor, subunit 2 | 1,56 |
| STM1777 | hemA | glutamyl tRNA reductase | 3,08 |
| STM2310 | menF | isochorismate synthase (isochorismate hydroxymutase 2), menaquinone biosynthesis | 1,90 |
| STM3056 | visC | putative monooxygenase | 1,59 |
| STM3057 | ubiH | 2-octaprenyl-6-methoxyphynol hydroxylase | 1,54 |
| STM3061 | ygfA | putative ligase | 1,69 |
| STM3104 | yggW | putative oxidase | 1,56 |
| STM3195 | ribB | 3,4 dihydroxy-2-butanone-4-phosphate synthase | 1,55 |
| STM3206 | folB | dihydroneopterin aldolase, also has dihydroneopterin triphosphate 2'-epimerase activity | 1,51 |
| STM3583 | acpT | putative Phosphopantetheinyl transferase | 1,77 |
| STM3725 | kdtB | phosphopantetheine adenylyltransferase | 1,66 |
| STM3875 | mioC | falavodoxin involved in biotin synthesis | 4,07 |
| STM4139 | coaA | pantothenate kinase | 1,56 |
| STM4164 | thiC | 5'-phosphoryl-5-aminoimidazole = 4-amino-5-hydroxymethyl-2-methylpyrimidine-P | 1,75 |
| **Drug/analog resistance/detoxification** | | | |
| STM0090 | ksgA | S-adenosylmethionine-6-N',N'-adenosyl (rRNA) dimethyltransferase; kasugamycin resistance | 2,07 |
| STM0376 | sbmA | putative ABC superfamily peptide-antibiotic transporter | 1,82 |
| STM0476 | acrA | acridine efflux pump | 1,62 |
| STM0477 | acrR | acrAB operon repressor (TetR/AcrR family) | 1,53 |
| STM1518 | marB | multiple antibiotic resistance protein | 2,04 |
| STM1519 | marA | transcriptional activator of defense systems (AraC/XylS family), multiple antibiotic resistance protein | 2,49 |
| STM1520 | marR | transcriptional repressor of marRAB operon, multiple antibiotic resistance protein | 2,74 |
| STM2221 | bcr | MFS family multidrug transport protein, bicyclomycin resistance protein | 2,69 |
| STM2814 | emrA | multidrug resistance secretion protein | 3,48 |
| STM2815 | emrB | putative MFS superfamily, multidrug transport protein | 2,86 |
| STM3117 | STM3117 | putative lactoylglutathione lyase | 2,04 |
| STM3205 | bacA | bacitracin resistance; possibly phosphorylates undecaprenol | 1,98 |
| STM3364 | yhcP | p-hydroxybenzoic acid efflux pump subunit AaeB | 1,81 |
| **Energy production and conversion** | | | |
| STM0564 | STM0564 | putative oxidoreductase | 1,56 |
| STM0858 | STM0858 | putative dehydrogenase (flavoproteins) | 1,88 |
| STM0956 | cydC | ABC superfamily (atp&memb), cytochrome-related transporter | 1,61 |
| STM1083 | yccX | putative phosphohydrolase | 2,04 |
| STM1458 | ydgM | putative alternative beta subunit of Na+-transporting NADH:ubiquinone oxidoreductase | 3,10 |
| STM1459 | STM1459 | putative oxidoreductase, inner membrane protein | 2,72 |
| STM1737 | tonB | energy transducer; uptake of iron, cyanocobalimin; sensitivity to phages, colicins | 1,67 |
| STM1788 | STM1788 | putative Ni/Fe-hydrogenase 1 b-type cytochrome subunit | 1,69 |
| STM3276 | yhbW | putative alkanal monooxygenase | 1,60 |
| STM4280 | nrfD | putative nitrate reductase, formate dependent | 1,58 |
| STM4355 | yjeS | putative Fe-S protein | 2,18 |
| **Inorganic ion transport and metabolism** | | | |
| STM0086 | kefC | CPA2 family, K+ efflux antiporter, glutathione-regulated | 1,77 |
| STM0425 | thiI | sulfur transfer protein (from cys to ThiS and from IscS to U8-tRNA) | 2,97 |
| STM0492 | ybaL | putative CPA2 family transport protein | 1,90 |
| STM0512 | sfbC | putative binding-protein-dependent transport systems inner membrane component | 1,57 |
| STM0667 | ybeX | putative CBS domain-containing protein | 1,78 |
| STM0758 | ybgR | putative CDF family transport protein | 1,53 |
| STM1084 | yccK | putative sulfite reductase, gamma subunit | 1,66 |
| STM1482 | ydgF | putative membrane transporter of cations and cationic drugs | 1,89 |
| STM1483 | ydgE | putative membrane transporter of cations and cationic drugs | 2,01 |
| STM1490 | STM1490 | putative chloride channel protein | 2,24 |
| STM1587 | yncD | paral putative outer membrane receptor | 1,80 |
| STM1741 | STM1741 | putative voltage-gated potassium channel | 2,22 |
| STM1743 | oppD | peptide/nickel transport system ATP-binding protein | 1,84 |
| STM1745 | oppB | peptide/nickel transport system permease protein | 1,71 |
| STM1891 | znuA | ABC superfamily (bind_prot) high affinity Zn transport protein | 1,87 |
| STM1892 | znuC | ABC superfamily (atp_bind) high affinity Zn transport protein | 1,67 |
| STM2408 | mntH | Nramp family, manganese/divalent cation transport prortein | 1,66 |
| STM2679 | yfjD | putative membrane protein | 2,21 |
| STM2783 | nxiA | putative nickel transporter | 2,20 |
| STM2798 | ygaP | putative rhodanese-related sulfurtransferase | 1,64 |
| STM2863 | sitC | Salmonella iron transporter: fur regulated | 2,83 |
| STM2864 | sitD | Salmonella iron transporter: fur regulated | 2,94 |
| STM3528 | STM3528 | putative periplasmic phosphate-binding protein | 1,55 |
| STM4324 | cutA | putative periplasmic divalent cation tolerance protein; cytochrome c biogenesis | 2,13 |
| STM4338 | sugE | putative DMT superfamily transport protein | 2,40 |
| **Lipid metabolism** | | | |
| STM0221 | uppS | undecaprenyl pyrophosphate synthetase (di-trans,poly-cis-decaprenylcistransferase) | 2,11 |
| STM0490 | aes | acetyl esterase | 1,77 |
| STM0812 | ybhO | cardiolipin (CL) synthase | 1,72 |
| STM1148 | ymdC | putative phospholipase | 1,60 |
| STM1350 | ydiD | homologue of a plant pathogenicity factor | 2,29 |
| STM1623 | STM1623 | putative carboxylesterase | 1,55 |
| STM2652 | pssA | phosphatidylserine synthase (CDP-diacylglycerol-serine O-phosphatidyltransferase) | 1,67 |
| **Nucleotide transport and metabolism** | | | |
| STM0533 | purK | phosphoribosylaminoimidazole carboxylase = AIR carboxylase, CO(2)-fixing subunit | 1,88 |
| STM0534 | purE | phosphoribosylaminoimidazole carboxylase = AIR carboxylase, catalytic subunit | 2,38 |
| STM1756 | purU | formyltetrahydrofolate hydrolase | 1,66 |
| STM1883 | purT | phosphoribosylglycinamide formyltransferase 2 | 1,73 |
| STM2121 | dcd | dUTPase | 1,51 |
| STM2122 | udk | uridine/cytidine kinase | 1,64 |
| STM2187 | yeiA | putative dihydropyrimidine dehydrogenase | 1,57 |
| STM2953 | pyrG | CTP synthetase | 2,49 |
| STM3733 | pyrE | orotate phosphoribosyltransferase | 1,64 |
| STM4176 | purH | bifunctional: phosphoribosylaminoimidazolecarboxamide formyltransferase; IMP cyclohydrolase | 2,11 |
| **Protein fate and modification** | | | |
| STM1484 | STM1484 | putative protease | 2,28 |
| STM1879 | ptrB | protease II | 1,58 |
| **Signal transduction** | | | |
| STM0053 | STM0053 | putative transcription regulator, histidine kinase for citrate | 1,68 |
| STM0398 | phoR | sensory kinase in two-component regulatory system with PhoB, regulates pho regulon | 1,97 |
| STM0468 | ylaB | putative diguanylate cyclase/phosphodiesterase domain 2 | 2,07 |
| STM1095 | copS | Copper resistance; histidine kinase | 1,52 |
| STM1283 | yeaJ | putative Methyl-accepting chemotaxis protein; Diguanylate cyclase/phosphodiesterase domain 1 | 2,36 |
| STM1285 | yeaG | putative Ser protein kinase | 1,58 |
| STM1344 | ydiV | putative Diguanylate cyclase/phosphodiesterase domain 1 | 1,58 |
| STM1660 | fnr | transcriptional regulation of aerobic, anaerobic respiration, osmotic balance (CRP family) | 1,59 |
| STM1766 | narX | sensory histidine kinase in two component regulatory system with NarL, senses nitrate/nitrite, regulates anaerobic respiration and fermentation | 2,02 |
| STM1827 | STM1827 | putative diguanylate cyclase/phosphodiesterase | 2,05 |
| STM1987 | STM1987 | putative inner membrane protein | 1,68 |
| STM2215 | rtn | putative membrane protein involved in resistance to lambda and N4 phages | 1,89 |
| STM2271 | rcsC | sensory histidine kinase in two-component regulatory system with RcsB, regulates colanic capsule biosynthesis | 1,77 |
| STM2503 | STM2503 | putative diguanylate cyclase | 1,76 |
| STM2564 | yfhK | putative sensory kinase in regulatory system | 1,65 |
| STM2580 | era | GTPase believed to be involved in coordination of cell cycle, energy metabolism, cell division | 1,92 |
| STM2637 | rseC | regulator of sigma E (sigma 24) factor | 2,00 |
| STM2638 | rseB | anti sigma E (sigma 24) factor, negative regulator | 2,58 |
| STM2672 | yfiN | putative diguanylate cyclase/phosphodiesterase | 3,37 |
| STM3522 | rtcR | sigma N (sigma 54)-dependent regulator of rtcBA expression (EBP familiy) | 1,67 |
| STM3615 | yhjK | putative Diguanylate cyclase/phosphodiesterase | 1,87 |
| STM3826 | torS | sensory kinase in multi-component regulatory system with TorR (regulator) and TorT (periplasmic sensor), regulates tor operon | 1,66 |
| STM4589 | creC | sensory kinase (alternative) in two-component regulatory system with CreB (or alternatively PhoB), senses catabolite repression, | 1,68 |
| **SPI1** | | | |
| STM2901 | STM2901 | putative cytoplasmic protein | 1,64 |
| STM2902 | STM2902 | putative cytoplasmic protein | 1,58 |
| **SPI2** | | | |
| STM1418 | ssaQ | secretion system apparatus protein SSAQ. (SW:SSAQ_SALTY); secretion system apparatus protein [Salmonella typhimurium LT2]. | 1,57 |
| STM1422 | ssaU | Secretion system apparatus: homology with YscU of the secretion system of Yersinia | 2,21 |
| **SPI3** | | | |
| STM3758 | fidL | putative inner membrane protein | 2,26 |
| STM3759 | marT | putative transcriptional regulatory protein | 2,89 |
| STM3764 | mgtC | Mg2+ transport protein | 2,07 |
| **SPI5** | | | |
| STM1087 | pipA | Pathogenicity island encoded protein: SPI3 | 3,50 |
| **Islands (not covered in other groups)** | | | |
| STM4311 | tnpA_6 | S. typhimurium transposase for insertion sequence element is200 (SW:T200_SALTY) | 1,51 |
| STM2766 | STM2766 | putative cytoplasmic protein [Salmonella typhimurium LT2]. | 1,51 |
| STM0716 | STM0716 | Paralog of E. coli recombinase involved in phase variation; regulator for fimA (AAC77268.1); Blastp hit to AAC77268.1 (200 aa), 60% identity in aa 9 - 192 | 1,52 |
| STM1015 | STM1015 | dnaC protein homolog (gi|7443681); Gifsy-2 prophage ATPase involved in DNA replication initiation [phage Gifsy-2]. | 1,53 |
| STM4492 | STM4492 | putative cytoplasmic protein [Salmonella typhimurium LT2]. | 1,54 |
| STM4196 | STM4196 | putative cytoplasmic protein [Salmonella typhimurium LT2]. | 1,54 |
| STM1561 | STM1561 | putative outer membrane or secreted lipoprotein [Salmonella typhimurium LT2]. | 1,57 |
| STM2767 | STM2767 | putative superfamily I DNA and RNA helicase [Salmonella typhimurium LT2]. | 1,57 |
| STM1638 | STM1638 | similar to E. coli orf, hypothetical protein (AAC73663.1); Blastp hit to AAC73663.1 (143 aa), 55% identity in aa 1 - 140; putative SAM-dependent methyltransferases [Salmonella typhimurium LT2]. | 1,58 |
| STM0292 | STM0292 | similar to E. coli rhsE protein in rhs element (AAC74538.1); Blastp hit to AAC74538.1 (682 aa), 44% identity in aa 426 - 541, 28% identity in aa 273 - 300; putative RHS-family protein [Salmonella typhimurium LT2]. | 1,58 |
| STM1532 | STM1532 | putative dehydrogenase protein [Salmonella typhimurium LT2]. | 1,59 |
| STM4575 | STM4575 | similar to E. coli putative transcriptional regulator (AAC76251.1); Blastp hit to AAC76251.1 (238 aa), 31% identity in aa 5 - 238; putative outer membrane protein [Salmonella typhimurium LT2]. | 1,63 |
| STM1629 | STM1629 | hypothetical protein | 1,63 |
| STM2743 | STM2743 | putative cytoplasmic protein [Salmonella typhimurium LT2]. | 1,66 |
| STM2616 | STM2616 | similar to antirepressor protein of phage P22; Gifsy-1 prophage [Salmonella typhimurium LT2]. | 1,68 |
| STM1673 | STM1673 | similar to E. coli orf, hypothetical protein (AAC75872.1); Blastp hit to AAC75872.1 (72 aa), 33% identity in aa 1 - 71; putative outer membrane lipoprotein [Salmonella typhimurium LT2]. | 1,75 |
| STM2627 | STM2627 | similar to cI protein in phage N; probable regulatory protein (gi|7467282); Gifsy-1 prophage protein [Salmonella typhimurium LT2]. | 1,78 |
| STM1251 | agsA | similar to E. coli heat shock protein (AAC76710.1); Blastp hit to AAC76710.1 (137 aa), 33% identity in aa 1 - 136; putative molecular chaperone (small heat shock protein) [Salmonella typhimurium LT2]. | 1,87 |
| STM3036 | STM3036 | putative inner membrane protein [Salmonella typhimurium LT2]. | 1,87 |
| STM4571 | STM4571 | Putative RBS for STM4571; RegulonDB:STMS1H004434 | 1,96 |
| STM0277 | STM0277 | Putative RBS for STM0277; RegulonDB:STMS1H000637 | 1,98 |
| STM3194 | STM3194 | putative disulfide isomerase [Salmonella typhimurium LT2]. | 1,98 |
| STM1266 | STM1266 | similar to E. coli putative transcriptional regulator (AAC74246.1); Blastp hit to AAC74246.1 (243 aa), 31% identity in aa 1 - 241; putative transcriptional regulator [Salmonella typhimurium LT2]. | 2,06 |
| STM0859 | STM0859 | similar to E. coli putative transcriptional regulator LYSR-type (AAC74667.1); Blastp hit to AAC74667.1 (297 aa), 28% identity in aa 1 - 264; putative LysR family transcriptional regulator [Salmonella typhimurium LT2]. | 2,16 |
| STM4574 | STM4574 | similar to E. coli putative transcriptional regulator (AAC76251.1); Blastp hit to AAC76251.1 (238 aa), 32% identity in aa 1 - 238; putative outer membrane protein [Salmonella typhimurium LT2]. | 2,17 |
| STM1267 | STM1267 | similar to E. coli orf, hypothetical protein (AAC74250.1); Blastp hit to AAC74250.1 (88 aa), 41% identity in aa 23 - 88; putative cytoplasmic protein [Salmonella typhimurium LT2]. | 2,21 |
| STM1250 | STM1250 | putative cytoplasmic protein [Salmonella typhimurium LT2]. | 2,22 |
| STM0266 | STM0266 | putative cytoplasmic protein [Salmonella typhimurium LT2]. | 2,25 |
| STM0293 | STM0293 | Putative RBS for STM0293; RegulonDB:STMS1H000653 | 2,33 |
| STM2625 | STM2625 | similar to dnaC homolog in S. typhimurium; dnaC protein homolog (gi|7443681); Gifsy-1 prophage protein [Salmonella typhimurium LT2]. | 2,53 |
| STM2624 | STM2624 | hypothetical protein (gi|7467246); Gifsy-1 prophage protein [Salmonella typhimurium LT2]. | 2,72 |
| STM1016 | STM1016 | S. typhimurium hypothetical protein (gi|7467246) | 2,90 |
| STM2623 | STM2623 | hypothetical protein 11 (gi|7467260); Gifsy-1 prophage protein [Salmonella typhimurium LT2]. | 3,02 |
| STM1243 | STM1243 | cold shock-like protein CSPH. (SW:CSPH_SALTY); homology with cold shock proteins [Salmonella typhimurium LT2]. | 9,62 |
| **Surface structure** | | | |
| STM0552 | fimW | negative regulator of type 1 fimbrial expression | 1,69 |
| STM1139 | csgG | putative transcriptional regulator in curly assembly/transport, 2nd curli operon | 2,45 |
| STM4572 | stjB | putative fimbrial usher protein | 1,68 |
| STM4573 | stjC | putative fimbrial chaparone protein | 1,52 |
| **Translation, ribosomal structure and biogenesis** | | | |
| STM0095 | rluA | 23S rRNA pseudouridylate 746 synthase | 1,81 |
| STM0185 | yadB | putative glutamyl t-RNA synthetase | 1,89 |
| STM0852 | yliG | putative Fe-S oxidoreductases family 1 | 1,68 |
| STM0882 | ybjF | putative tRNA (uracil-5-)-methyltransferase | 1,79 |
| STM0953 | infA | protein chain initiation factor IF-1 | 2,35 |
| STM1187 | rluC | 23S rRNA pseudouridylate synthase | 2,17 |
| STM1234 | trmU | tRNA (5-methylaminomethyl-2-thiouridylate)-methyltransferase | 3,44 |
| STM1449 | tyrS | tyrosine tRNA synthetase | 1,62 |
| STM1719 | yciL | putative ribosomal large subunit pseudouridine synthase | 1,88 |
| STM2222 | rsuA | 16S rRNA pseudouridylate 516 synthase | 3,28 |
| STM2545 | STM2545 | putative rRNA methylase | 1,96 |
| STM2648 | yfiF | putative tRNA/rRNA methyltransferase | 1,51 |
| STM2928 | ygbO | tRNA pseudouridine synthase D | 1,51 |
| STM3220 | ygjO | paral putative methyltransferase | 1,75 |
| STM3288 | yhbC | ribosome maturation factor RimP | 3,40 |
| STM3298 | yhbY | putative RNA-binding protein containing KH domain | 2,15 |
| STM3304 | rplU | 50S ribosomal subunit protein L21 | 1,98 |
| STM3370 | cafA | RNase G | 1,52 |
| STM3402 | yrdC | putative translation factor | 1,82 |
| STM3497 | yrfH | heat shock protein, predicted small RNA-binding protein | 1,81 |
| STM3682 | selB | selenocysteinyl-tRNA-specific translation factor | 1,52 |
| STM4344 | yjeA | putative pyruvate oxidase (lysyl-tRNA synthetase) | 1,83 |
| **Transcription** | | | |
| STM0606 | ybdO | putative transcriptional regulator, LysR family | 1,64 |
| STM0652 | STM0652 | putative sigma-54 dependent transcriptional regulator | 1,60 |
| STM0682 | nagC | transcriptional repressor of nag (N-acetylglucosamine) operon (NagC/XylR family) | 1,79 |
| STM0821 | dinG | LexA regulated (SOS) repair enzyme | 1,85 |
| STM0859 | STM0859 | putative transcriptional regulator, LysR family | 2,16 |
| STM0869 | STM0869 | paral putative regulator (TetR/Acr family) | 3,25 |
| STM0952 | STM0952 | putative transcriptional regulator, lysR family | 1,92 |
| STM1265 | STM1265 | putative response regulators consisting of a CheY-like receiver domain and a HTH DNA-binding domain | 1,78 |
| STM1437 | ydhM | putative transcriptional repressor (TetR/AcrR family) | 2,58 |
| STM1487 | ynfL | putative transcriptional regulator, LysR family | 1,58 |
| STM1510 | ydfH | putative regulatory protein, gntR family | 1,50 |
| STM1588 | yncC | putative regulatory protein, gntR family | 1,98 |
| STM1774 | sirC | Regulation of invasion genes | 2,58 |
| STM1821 | yoaA | putative DNA helicase | 1,61 |
| STM2201 | yeiE | putative transcriptional regulator, LysR family | 1,52 |
| STM2420 | xapR | regulator for XapA (LysR family) | 2,62 |
| STM2544 | yfhP | believed to be involved in assembly of Fe-S clusters | 2,08 |
| STM2575 | STM2575 | putative transcriptional regulator, LysR family | 1,61 |
| STM2581 | rnc | RNase III, ds RNA | 2,00 |
| STM2644 | yfiE | putative transcriptional regulator, LysR family | 1,90 |
| STM2797 | STM2797 | putative regulatory protein, arsR family | 2,27 |
| STM2813 | emrR | transcriptional repressor of emrAB operon (MarR family) | 1,76 |
| STM2859 | fhlA | formate hydrogen-lyase transcriptional activator for fdhF, hyc and hyp operons (EBP family) | 1,91 |
| STM2955 | STM2955 | putative transcriptional regulators containing the CopG/Arc/MetJ DNA-binding domain and a metal-binding domain | 1,53 |
| STM2979 | fucR | positive regulator of the fuc operon (DeoR family) | 2,00 |
| STM2982 | gcvA | regulator of gcv operon (LysR family) | 1,96 |
| STM3064 | iciA | inhibitor of replication initiation, also transcriptional regulator of dnaA and argK (LysR family) | 2,28 |
| STM3098 | STM3098 | putative transcriptional regulator | 1,62 |
| STM3163 | yqhC | putative transcriptional regulator (AraC/XylS family) | 1,81 |
| STM3299 | greA | transcription elongation factor, cleaves 3' nucleotide of paused mRNA | 1,87 |
| STM3648 | yiaG | putative transcriptional regulator | 2,96 |
| STM3662 | xylR | xylose operon regulatory protein (AraC/XylS family) | 1,99 |
| STM3741 | rpoZ | RNA polymerase, omega subunit | 1,64 |
| STM3834 | STM3834 | putative transcriptional regulator, LysR family | 2,56 |
| STM4237 | lexA | SOS response regulator, transcriptional repressor (LexA family) | 1,67 |
| STM4287 | phnO | putative regulator in phn operon | 1,77 |
| STM4337 | ecnR | putative bacterial regulatory protein, luxR family | 3,03 |
| STM4367 | yjeB | putative negative regulator | 1,54 |
| **Unknown function** | | | |
| STM0098 | STM0098 | putative secreted protein | 1,51 |
| STM0105 | yabI | putative DedA family, membrane protein | 2,49 |
| STM0312 | yafK | putative periplasmic protein | 1,72 |
| STM0837 | ybiS | putative periplasmic protein | 2,76 |
| STM0940 | ybjX | homologue of virK | 2,19 |
| STM1215 | ycfS | putative periplasmic protein | 1,58 |
| STM1229 | ycfD | putative cytoplasmic protein | 1,98 |
| STM1282 | yeaK | putative cytoplasmic protein | 2,09 |
| STM1284 | yeaH | putative cytoplasmic protein | 2,02 |
| STM1345 | ydiU | putative cytoplasmic protein | 1,51 |
| STM1701 | yciW | putative cytoplasmic protein | 1,82 |
| STM1834 | yebN | putative YebN family transport protein | 2,53 |
| STM1848 | yebS | putative inner membrane protein | 1,69 |
| STM2227 | yejL | putative cytoplasmic protein | 1,83 |
| STM2520 | yfgL | putative serine/threonine protein kinase | 1,61 |
| STM2981 | ygdD | putative small membrane protein | 1,90 |
| STM3065 | yggE | putative periplasmic immunogenic protein | 1,68 |
| STM3092 | sprT | putative cytoplasmic protein | 2,01 |
| STM3153 | yqhA | putative membrane-associated protein | 1,66 |
| STM3226 | yqjA | putative DedA family, membrane protein | 1,69 |
| STM3230 | yqjE | putative inner membrane protein | 1,74 |
| STM3232 | yqjF | putative membrane-associated protein | 1,55 |
| STM3270 | yhbP | putative cytoplasmic protein | 1,84 |
| STM3467 | yhfK | putative inner membrane protein | 1,63 |
| STM3706 | yigQ | putative periplasmic protein | 1,55 |
| STM4169 | yjaG | putative cytoplasmic protein | 1,70 |
| STM4288 | phnB | putative cytoplasmic protein | 2,45 |
| STM4516 | yjiN | putative inner membrane protein | 1,60 |
